# Enhanced Photocatalytic H_2_O_2_ Production on Terpyridine‐Based Acrylonitrile‐Linked Covalent Organic Frameworks with Asymmetric Localized Electron Distribution

**DOI:** 10.1002/adma.73821

**Published:** 2026-06-20

**Authors:** Qinglan Zhao, Mengmeng Ce, Hengtao Lei, Tianyun Jin, Zhipeng Xie, Yushen Liu, Shengyi Yang, Xinwen Ou, Mohammad Farhadpour, Song‐Zhu Kure‐Chu, Xuan‐He Liu, Liang Zhang, Jimmy C. Yu

**Affiliations:** ^1^ Department of Chemical and Biological Engineering The Hong Kong University of Science and Technology Kowloon Hong Kong China; ^2^ School of Science China University of Geosciences (Beijing) Beijing China; ^3^ Key Laboratory of Precision and Intelligent Chemistry Hefei National Research Center for Physical Sciences at the Microscale School of Chemistry and Materials Science University of Science and Technology of China Hefei China; ^4^ Department of Chemistry The Hong Kong University of Science and Technology Kowloon Hong Kong China; ^5^ Department of Chemistry The Chinese University of Hong Kong Hong Kong China; ^6^ School of Materials Science and Engineering Southeast University Nanjing China

**Keywords:** covalent organic frameworks, hydrogen peroxide, oxygen reduction reaction, photocatalysis

## Abstract

Covalent organic frameworks (COFs) serve as promising photocatalysts for sustainable photosynthesis of H_2_O_2_ from water and air due to their well‐defined architecture and precisely controllable structure. The key to achieving high H_2_O_2_ production rates lies in the efficient adsorption of O_2_ and effective charge transfer over COFs. In this work, we have studied a terpyridine‐based acrylonitrile‐linked COF (TPy‐acr COF) with optimum localized electron distribution and enhanced charge transfer for two‐electron transfer oxygen reduction toward H_2_O_2_ production. The electron‐deficient pyridine nitrogen atoms in the terpyridine unit induce an asymmetric electron distribution with localized electron density at the adjacent carbon atoms, while the electron‐withdrawing acrylonitrile linkage increases the electron distribution asymmetry of the entire framework. Consequently, the high electron‐density carbon atoms in the terpyridine units of TPy‐acr COF show strong binding for O_2_ and fast charge transfer to the key intermediate ^*^OOH. Remarkably, the TPy‐acr COF enabled a high production rate of 4.85 mmol g^−1^ h^−1^ for H_2_O_2_ directly from water and air. An outdoor scale‐up experiment shows an average H_2_O_2_ production rate of 1.14 mmol g^−1^ h^−1^ over TPy‐acr COF using water in ambient air under natural sunlight, demonstrating practical application potential with sustainability, simplicity, and scalability.

## Graphical abstract

Asymmetric localized electron distribution has been optimized in covalent organic frameworks to enhance ^*^O_2_ adsorption and facilitate charge transfer, boosting photocatalytic two‐electron transfer oxygen reduction toward H_2_O_2_ production from water and air.

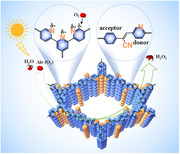

## 1 Introduction

Hydrogen peroxide production as a billion‐dollar industry has wide applications in various sectors including pharmaceuticals, environmental services, and chemical manufacturing. Currently, more than 95% of industrial H_2_O_2_ production relies on the carbon‐ (0.25 mol of CO_2_ per mol of H_2_O_2_ produced) and energy‐intensive (aggregate consumption up to 17.6 kWh kg^−1^ H_2_O_2_) anthraquinone process associated with steam‐methane reforming for H_2_ feed [1, 2, 3]. The global market of H_2_O_2_ is projected to reach 7.71 Mt by 2031 [4], generating an estimated energy consumption of approximately 135.7 MWh. Therefore, it is essential to explore efficient and sustainable alternative approaches.

Photosynthesis of H_2_O_2_ directly from pure water and O_2_ through oxygen reduction reaction (ORR) using renewable solar energy presents an ideal pathway. Since Hoffmann and his colleagues first demonstrated the aqueous suspension of TiO_2_, ZnO, and desert sand for photocatalytic H_2_O_2_ production in 1988 [5], various inorganic metal oxide semiconductors have been applied for the photosynthesis of H_2_O_2_ via ORR [6, 7, 8]. However, due to the strong affinity between metal ions and H_2_O_2_, the surface‐adsorbed H_2_O_2_ is prone to oxidation and decomposition by photogenerated holes, significantly inhibiting both the activity and selectivity for H_2_O_2_ production [9, 10, 11]. Therefore, metal‐free catalysts are increasingly desirable for the photosynthesis of H_2_O_2_.

Covalent organic frameworks (COFs), recognized as a new class of metal‐free semiconductor materials with a well‐defined donor–acceptor structure, highly delocalized π‐electrons, and high surface area, have garnered substantial interests as photocatalysts for H_2_O_2_ production due to their adjustable bandgap, efficient exciton migration, and fast mass transport. However, most reported symmetric 2D COFs lack sufficient charge transfer with limited localized bonding sites for oxygen species, resulting in a low yield of photosynthetic H_2_O_2_ [12]. Constructing highly localized electron density by manipulating the molecular structure has been demonstrated as an effective strategy for enhancing the adsorption interaction with O_2_ and favoring the binding of the key intermediate ^*^OOH, accelerating the direct photosynthesis of H_2_O_2_ from oxygen, water, and sunlight [13, 14].

**FIGURE 1:**
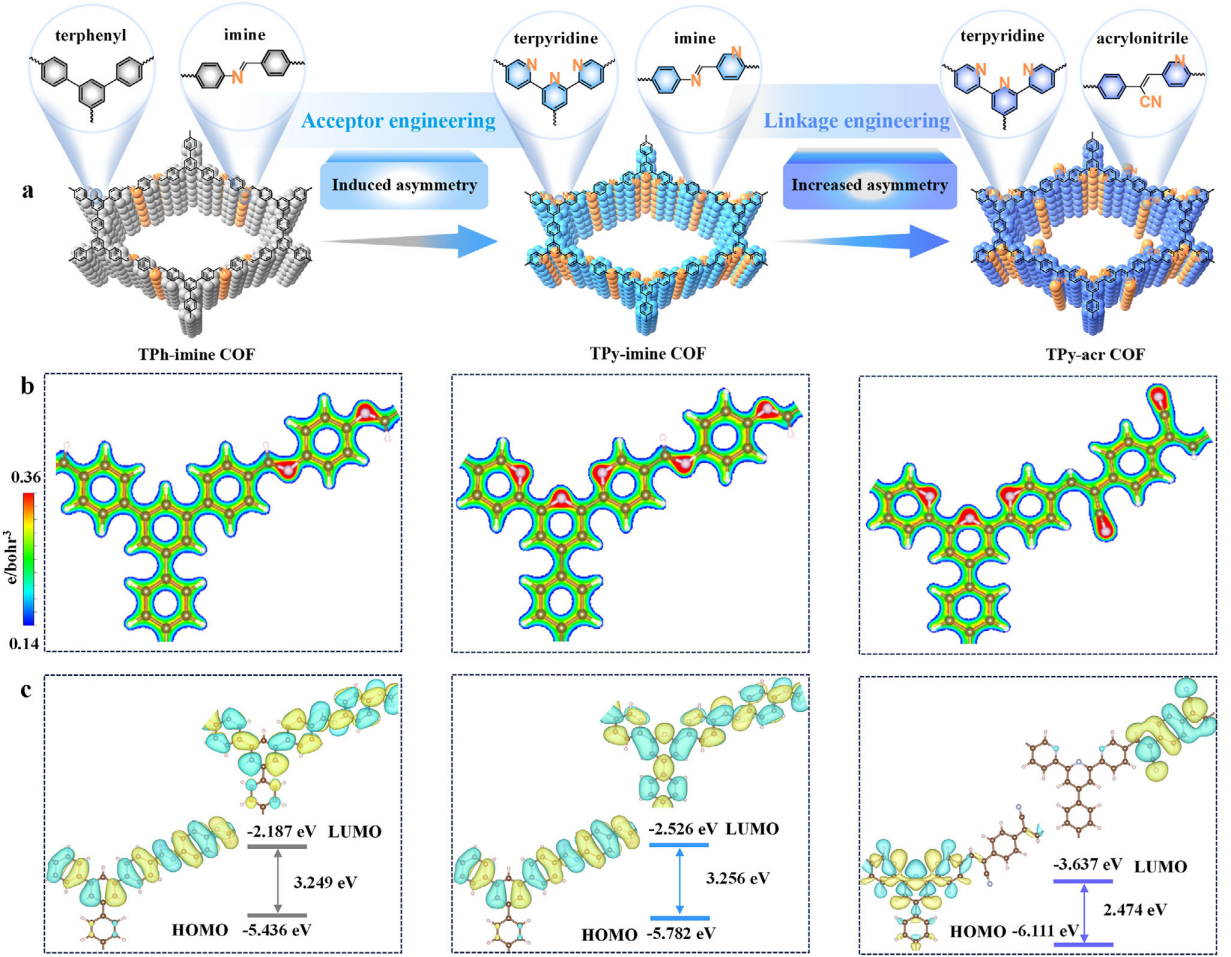
Molecular design of COFs. (a) Schematic of molecular engineering of COFs through tailoring the donor–acceptor units and linkages. (b) Charge distribution map of TPh‐imine COF, TPy‐imine COF, and TPy‐acr COF. (c) Distribution of HOMO and LUMO with the corresponding orbital energy levels of TPh‐imine COF, TPy‐imine COF, and TPy‐acr COF.

Guided by the first‐principles simulations, we selected a series of molecular‐engineered COFs featuring dense donor–acceptor lattices with engineered acceptors and linkages for enhanced asymmetric electron distribution to promote the binding of O_2_ and facilitate charge transfer to the adsorbed oxygen species. We started with our terpyridine (TPy)‐based COFs, which have been successfully applied in photodynamic therapy due to their exceptional light absorption and photostability [15]. Specifically, as shown in Figure 1a, we employed TPy with three electron‐deficient pyridine nitrogen atoms to induce the asymmetric electron distribution and used an acrylonitrile (acr) linkage to further optimize the asymmetry of the entire framework, favoring the adsorption of O_2_ and facilitating charge transfer. The carbon atoms neighboring nitrogen atoms have increased local electron density with an asymmetrical structure in the obtained TPy‐acr COF according to our simulation (Figure 1b), which can facilitate the injection of photogenerated electrons into the π^*^ anti‐bonding orbitals of O_2_ [13], significantly promoting the adsorption of O_2_ and oxygen intermediates. The introduction of the acrylonitrile linkage further increases the electron distribution asymmetry of TPy‐acr COF to facilitate the electron transfer between the (Z)‐2,3‐diphenylacrylo‐nitrile (DPAN) unit and TPy unit within the framework, which may also benefit charge transfer for improved reaction kinetics. As shown in Figure 1c, all COFs are expected to possess suitable theoretical bandgaps as photocatalysts, ranging from 2.474 to 3.256 eV. In addition, the TPy‐acr COF shows spatial distribution of the highest occupied molecular orbital (HOMO) and the lowest unoccupied molecular orbital (LUMO). On the contrary, terphenyl (TPh)‐imine COF and TPy‐imine COF host a delocalized HOMO and LUMO distribution almost across the entire framework. The primarily concentrated HOMO distribution within the TPy unit of the TPy‐acr COF suggests the electron‐donating propensity of the TPy unit induced by the electron‐withdrawing acrylonitrile linker. The spatial distribution of HOMO and LUMO on donor and acceptor units facilitates the charge transfer within the entire framework, thereby favoring the binding and activation of O_2_ onto this unit for subsequent reduction reaction toward H_2_O_2_ formation. As a result, the TPy‐acr COF photocatalyst enabled photosynthesis of H_2_O_2_ directly from pure water and air with a high production rate of 4.85 mmol g^−1^ h^−1^, apparent quantum yield of 2.6%–2.4% at 400–450 nm in the absence of metals and sacrificial agents. Furthermore, an average production rate of 1.14 mmol g^−1^ h^−1^ was achieved in the scale‐up production of H_2_O_2_ over TPy‐acr COF in open air under natural sunlight in an outdoor experiment, demonstrating potential for scalable practical application.

## 2 Results and Discussion

**FIGURE 2:**
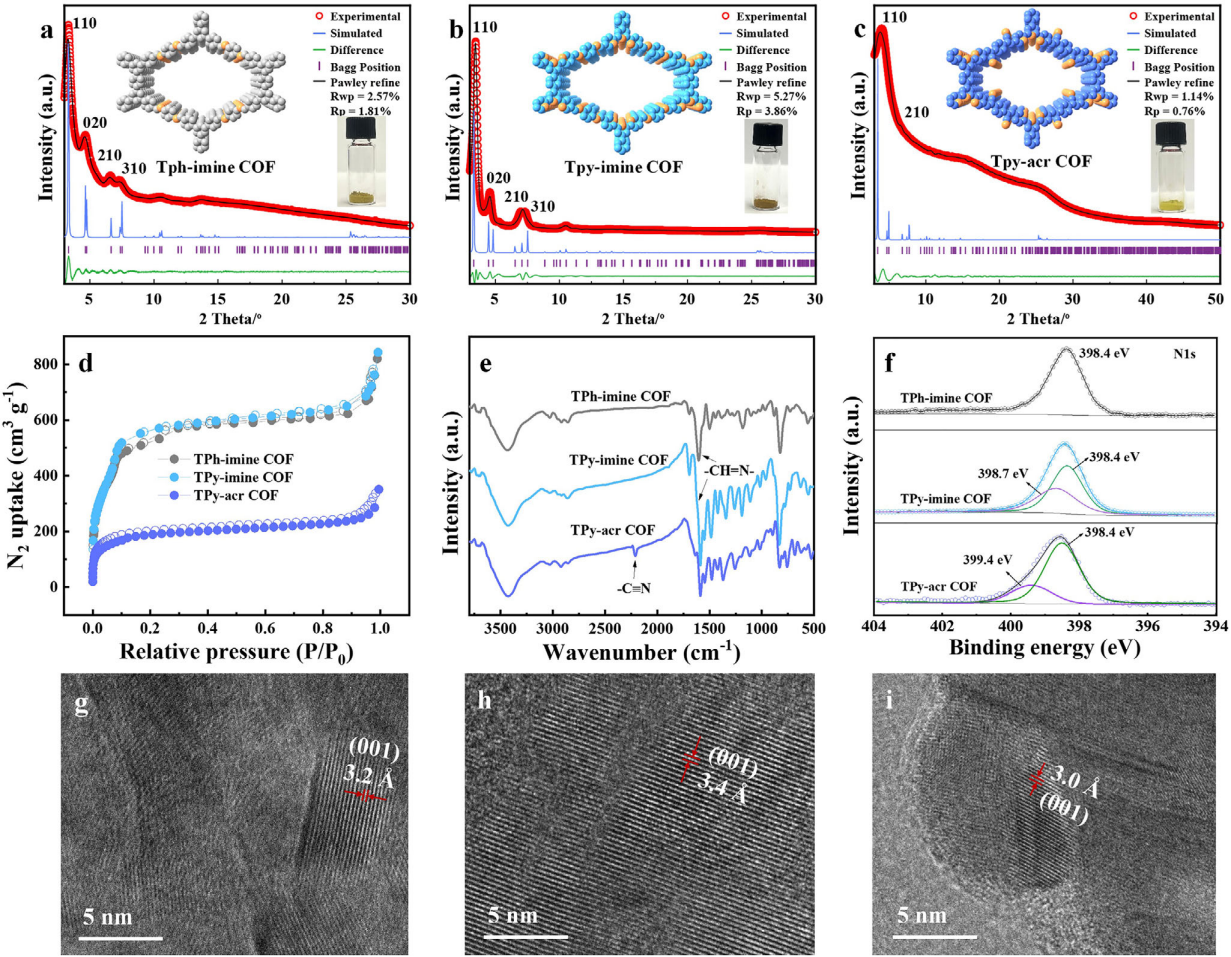
Structural characterization and bonding information of COFs. (a–c) Experimental XRD patterns (red circle), Pawley refined patterns (black curve), Bragg position (purple line), simulated sync AA stacking pattern (blue curve and the inset), and their differences (green curve) of TPh‐imine COF (a), TPy‐imine COF (b), and TPy‐acr COF (c). (d) N_2_ adsorption–desorption isotherms (solid circle, adsorption; open circle, desorption). (e) FTIR spectra. (f) N1s XPS spectra and the corresponding deconvolution. (g–i) High‐resolution TEM images of TPh‐imine COF (g), TPy‐imine COF (h), and TPy‐acr COF (i).

The general construction of these COF materials, including TPh‐imine COF, TPy‐imine COF, and TPy‐acr COF, was conducted according to our previous reports (see Methods for details) [15, 16]. The crystalline structures of these COFs (Figure 2a–c) were revealed by the powder X‐ray diffraction (XRD) patterns together with theoretical structural modeling. All COFs exhibited distinct characteristic peaks: the TPh‐imine COF at 3.35°, 4.62°, 6.58°, 7.34°, respectively, corresponding to the (110), (020), (210), and (310) facets; the TPy‐imine COF at 3.49°, 4.56°, 6.96°, 7.34°, and 10.50°, respectively, corresponding to the (110), (020), (130), (310), and (240) facets; and the TPy‐acr COF at 3.75°, and 6.67°, respectively, corresponding to the (110) and (210) facets. Structural reconstruction (blue curve) with sync AA stacking mode followed by Pawley refinement (black curve) produced unit cell parameters of *a* = 3.17 nm, *b* = 3.80 nm, *c* = 3.51 Å, α = β = γ = 90° for TPh‐imine COF, *a* = 3.72 nm, *b* = 4.01 nm, *c* = 3.51 Å, α = β = γ = 90° for TPy‐imine COF, and a = 3.65 nm, b = 3.88 nm, c = 3.51 Å, α = β = γ = 90° for TPy‐acr COF. The low Rwp and Rp values (Rwp = 2.57%, Rp = 1.81% for TPh‐imine COF, Rwp = 5.27%, Rp = 3.86% for TPy‐imine COF, and Rwp = 1.14%, Rp = 0.76% for TPy‐acr COF) indicate that the experimental results match well with the refinement data.

Consistent with the sync AA stacking mode, the pore properties of the synthesized COFs were also confirmed by the nitrogen adsorption–desorption measurements at 77 K. As shown in Figure 2d, all COFs present a type I isotherm, indicating microporosity as the main pore distribution. The surface areas of TPh‐imine COF, TPy‐imine COF, and TPy‐acr COF are 1466.1, 1519.3, and 568.8 m^2^ g^−1^, respectively, according to the Brunauer–Emmett–Teller analysis. In addition, the pore sizes are mainly distributed at 2.35, 2.35, and 1.85 nm for TPh‐imine COF, TPy‐imine COF, and TPy‐acr COF, respectively, by applying the non‐local density functional theory (Figure S1a–c and Table S1).

The chemical bonding information was revealed using Fourier transform infrared (FTIR) spectroscopy and X‐ray photoelectron spectroscopy (XPS). The FTIR spectra of TPh‐imine COF and TPy‐imine COF show a stretching vibration signal at around 1605 cm^−1^ corresponding to the imino group (─CH═N─), while the one appearing at 2212 cm^−1^ can be assigned to the cyano group (─C≡N) of TPy‐acr COF (Figure 2e). The N1s XPS spectrum of TPh‐imine COF in Figure 2f displays a band at 398.4 eV belonging to the imino nitrogen. However, for TPy‐imine COF and TPy‐acr COF, the N1s XPS spectra can be fitted into two subpeaks approximately at 398.4 and 398.7 eV attributed to nitrogen from imine linkage and pyridine for TPy‐imine COF, while 398.4 and 399.4 eV can be assigned to imino nitrogen and cyano nitrogen for TPy‐acr COF, respectively, further confirming the successful synthesis of these COFs. High‐resolution transmission electron microscopy (TEM) images (Figure 2g–i) present parallel arrays of interlayers in all COF samples, further confirming their excellent crystallinity. The lattice fringe spacings of 3.2, 3.4, and 3.0 Å can be attributed to the interlayer distance of their (001) crystal plane.

**FIGURE 3:**
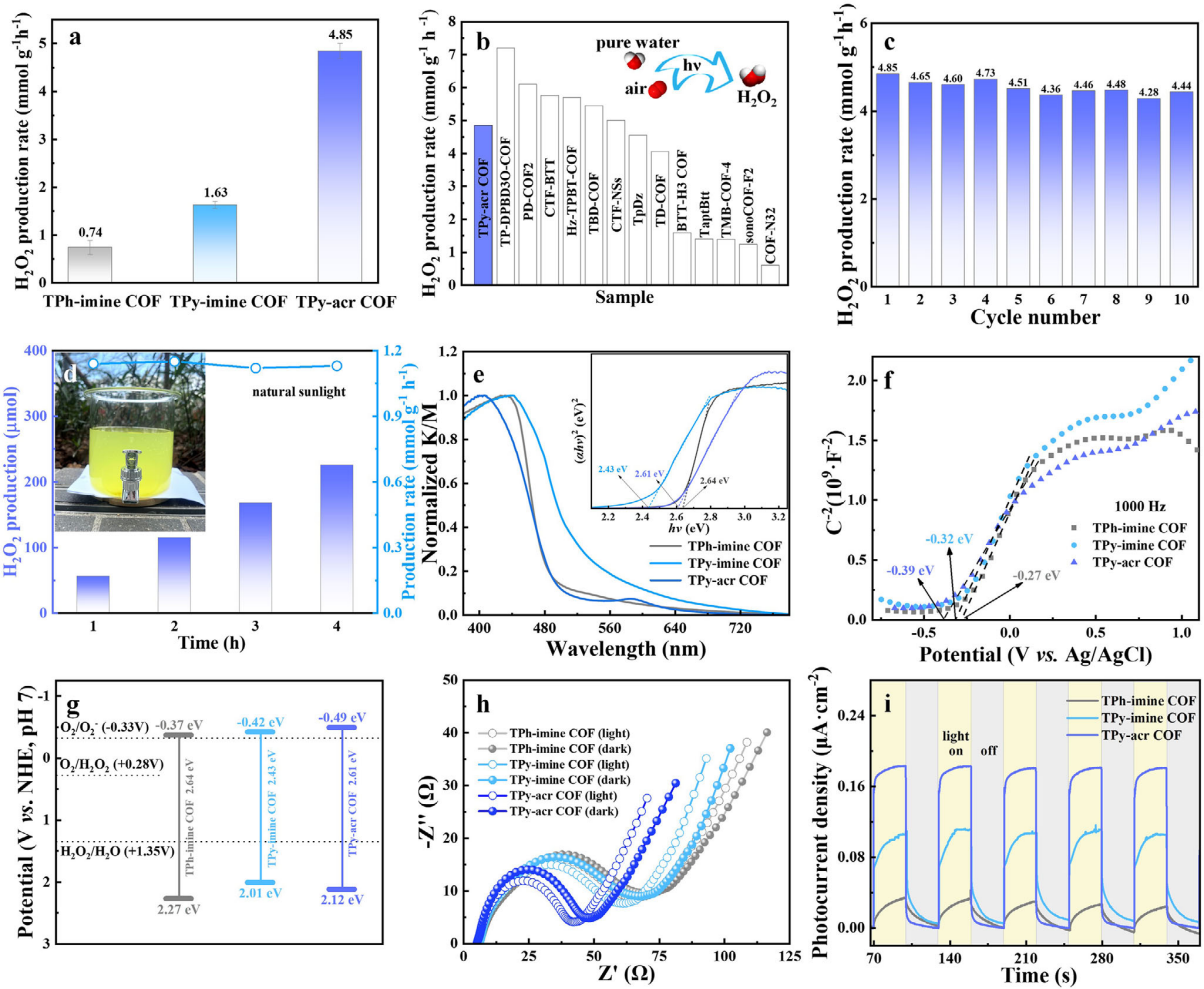
Photosynthesis of H_2_O_2_ directly from water and air. (a) Photocatalytic production rates of H_2_O_2_ over TPh‐imine COF, TPy‐imine COF, and TPy‐acr COF. (b) Comparison of H_2_O_2_ production rates on TPy‐acr COF with recently reported COF photocatalysts [17, 18, 19, 20, 21, 22, 23, 24, 25, 26, 27, 28, 29]. (c) The cyclic stability of TPy‐acr COF toward H_2_O_2_ production. (d) Photocatalytic performance of producing H_2_O_2_ using 75 mg of TPy‐acr COF catalyst in 1.5 L of pure water under natural sunlight, with the inset showing a digital image of the outdoor experiment at the 2^nd^ h (∼100 mW cm^−2^, 27°C–30°C, from 10:00 to 14:00, on 31 Oct 2025 in Hong Kong). (e) Solid‐state UV–vis spectra with the inset of Tauc plots (values with arrows indicate the optical bandgaps). (f) Mott–Schottky curves. (g) Experimental energy band alignments compared to the theoretical reduction potentials (V vs. NHE at pH = 7) of O_2_/O_2·_^−^, O_2_/H_2_O_2_, and H_2_O_2_/H_2_O. (h) Electrochemical impedance spectra (open circle, under light irradiation; solid circle, in the dark). (i) Transient photocurrent density measurements.

Visible‐light‐driven synthesis of H_2_O_2_ from pure water and air was carried out under light irradiation (350–780 nm) in a batch cell after optimizing catalyst dosage (Figure S2). The time‐dependent production of H_2_O_2_ for TPy‐acr COF indicates that the concentration of H_2_O_2_ quickly increased and exhibits an enhanced photocatalytic H_2_O_2_ yield of 9.47 µmol within 2 h of light irradiation, which is significantly higher than that of TPh‐imine COF (1.28 µmol) and TPy‐imine COF (3.09 µmol) (Figure S3). As shown in Figure 3a, TPh‐imine COF presents a low average H_2_O_2_ production rate of 0.74 mmol g^−1^ h^−1^. Upon the replacement of the *m*‐terphenyl unit by a TPy unit with induced asymmetric electron distribution around the imine bonds, TPy‐imine COF displays a notable increase in H_2_O_2_ production as high as 1.63 mmol g^−1^ h^−1^. After further linkage engineering by the introduction of an acrylonitrile group, TPy‐acr COF exhibits the highest production rate of 4.85 mmol g^−1^ h^−1^, which may benefit from the facilitated charge transfer between donor and acceptor units within the asymmetric framework. This performance stands out among most of the state‐of‐the‐art COF photocatalysts for H_2_O_2_ production from pure water and air (Figure 3b and Table S2) [17, 18, 19, 20, 21, 22, 23, 24, 25, 26, 27, 28, 29]. In addition to the high catalytic activity, TPy‐acr COF also exhibits a stable production rate after consecutive 10 photocatalytic cycles (Figure 3c). The well‐maintained morphology (Figure S4) and sharp XRD peaks of TPy‐acr COF (Figure S5) demonstrated its excellent stability for H_2_O_2_ production. Furthermore, the apparent quantum yield (AQY) of TPy‐acr COF exhibits a strong dependence on its absorption spectrum, with AQY of 2.6%–2.4% at 400–450 nm (Figure S6). To evaluate the potential of practical application, the performance of TPy‐acr COF was studied in open air under natural sunlight irradiation (Figure 3d). A nearly linear growth of H_2_O_2_ accumulation with an average production rate of 1.14 mmol g^−1^ h^−1^ was observed in a continuous 4 h outdoor experiment, further confirming the excellent light harvest capability of the TPy‐acr COF catalyst. Considering that photocatalytic H_2_O_2_ production is an outcome of combined formation and decomposition of H_2_O_2_ [7], a H_2_O_2_ decomposition experiment on TPy‐imine COF was also performed with an Ar‐saturated solution containing 2 mM H_2_O_2_. Less than 2% of H_2_O_2_ decomposed after 2 h illumination, further indicating the suitability of this catalyst for H_2_O_2_ photosynthesis (Figure S7).

Ignorable amounts of H_2_O_2_ were detected in N_2_‐saturated solution and under dark for all three COFs (Figure S8), which suggests that the produced H_2_O_2_ came from photocatalytic reduction of O_2_ in air rather than water oxidation reaction (WOR). The successful detection of ^18^O_2_ in the isotope‐labelling experiment using H_2_^18^O as water resource also indicates the 4e^−^ WOR pathway as similarly reported (Figure S9) [17]. Rotating ring‐disk electrode (RRDE) measurements were further conducted to examine the 4e^−^ or 2e^−^ WOR pathway at the generated holes during the photocatalytic process. As shown in Figure S10, the incremental disk currents at potentials above ∼1.3 V indicate that water oxidation occurs at the rotating disk electrode for all COF samples. The negligible oxidation currents on the ring electrode suggest the absence of H_2_O_2_ produced via the 2e^−^ WOR process (Figure S10a). In addition, the reduction currents occurring at the ring electrode were caused by the reduction of O_2_, further demonstrating the generation of O_2_ via the 4e^−^ WOR process (Figure S10b). Therefore, the H_2_O_2_ detected in the pure water system was produced via the photocatalytic 2e^−^ ORR process on COFs.

Subsequently, the optical and electronic properties of the synthesized COFs were also investigated. All three COFs show substantial photon‐harvesting response in the visible‐light range (Figure 3e), with the corresponding medium optical bandgaps of 2.64, 2.43, and 2.61 eV derived from Tauc plots (Figure 3e inset) for TPh‐imine COF, TPy‐imine COF, and TPy‐acr COF, respectively. As anticipated, these measured bandgap values are also within the range of our calculated bandgaps. According to the Mott−Schottky measurements (Figure 3f), the flat band potentials (V_fb_) of TPh‐imine COF, TPy‐imine COF, and TPy‐acr COF were determined to be −0.27, −0.32, and −0.39 V (vs. Ag/AgCl), respectively. By further integrating the optical bandgaps and Mott–Schottky plots, the conduction band (CB) and valence band (VB) levels were estimated for all COFs. As shown in Figure 3g, the TPy‐acr COF possesses the most negative CB level at −0.49 V (vs. normal hydrogen electrode (NHE) at pH = 7) compared to the other two COFs. The upshifted CB position can result in higher photoreduction ability of the TPy‐acr COF to drive the ORR toward forming H_2_O_2_ via the superoxide anion intermediate (O_2·_^−^, −0.33 V vs. NHE). Furthermore, these COFs show a smaller charge transfer resistance under light irradiation than in the dark due to the generation of photogenerated carriers [30], with TPy‐acr COF exhibiting the lowest one (Figure 3h). Consequently, the highest photocurrent density was achieved by the TPy‐acr COF (Figure 3i), indicating the greatest efficiency of photoinduced charge carrier generation and charge transfer. To further elucidate the behavior of photogenerated charge carriers, steady‐state photoluminescence (PL) spectroscopy and time‐resolved photoluminescence (TRPL) spectroscopy were also employed (Figure S11 and Table S3). Consistent with the photocurrent density results, higher fluorescence intensity of TPy‐acr COF indicates that the radiative recombination of photogenerated electron–hole pairs dominates the main process in the TPy‐acr COF photocatalyst, while the other two samples with much lower fluorescence intensities are dominated by non‐radiative recombination [31, 32]. The TRPL results further explain its favorable charge carrier dynamic performance. The longest average lifetime of TPy‐acr COF indicates that the photogenerated charges can have more chances to participate in the photocatalytic reaction [33].

**FIGURE 4:**
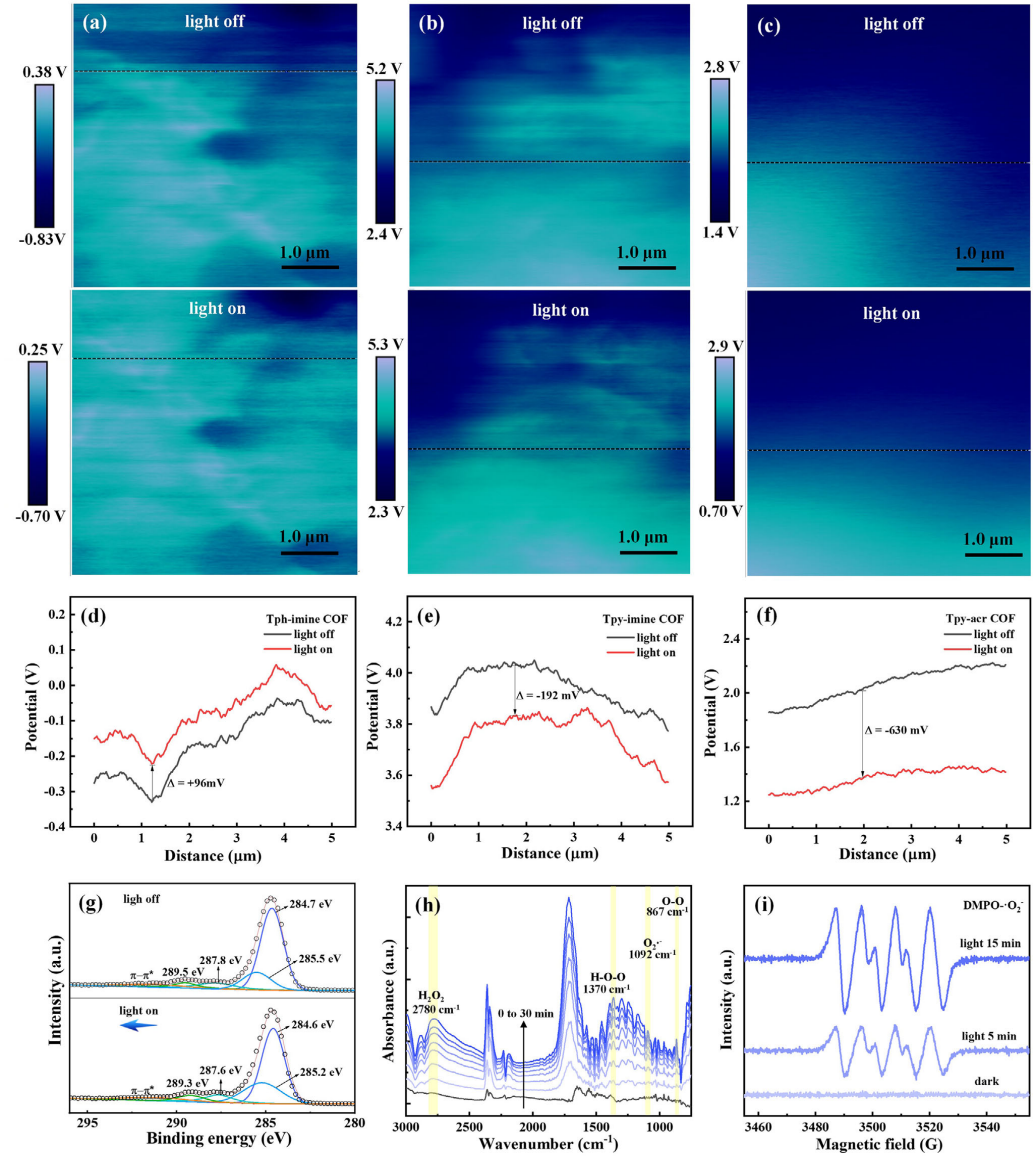
In situ characterizations. (a–c) In situ KPFM mapping of TPh‐imine COF (a), TPy‐imine COF (b), and TPy‐acr COF (c) with light illumination off and on. (d–f) The corresponding potential curves of TPh‐imine COF (d), TPy‐imine COF (e), and TPy‐acr COF (f) during in situ KPFM measurements. (g) In situ C1s XPS spectra of TPy‐acr COF with light illumination off and on. (h) Time‐dependent in situ DRIFTS spectra of TPy‐acr COF under light illumination with O_2_ flowing. (i) DMPO spin‐trapping ESR spectra of TPy‐acr COF for measuring O_2_∙^−^ in dark and light.

To reveal the charge carrier dynamics during the photocatalytic process and investigate the reaction pathway toward H_2_O_2_ formation, a series of in situ characterizations were conducted. As shown in Figure 4a–f, in situ Kelvin probe force microscopy (KPFM) measurements were performed to monitor the real‐time electron transfer behavior and surface potential variations. Except for TPh‐imine COF, showing a positive shift in surface potential of 96 mV under light illumination, both TPy‐imine COF and TPy‐acr COF present a negative shift in surface potential of 192 and 630 mV, respectively. The negative shift in surface potential resulted from an increased number of photo‐generated electrons transferred to the surface of TPy‐imine COF and TPy‐acr COF under light illumination [34]. The larger shift in surface potential of TPy‐acr COF suggests more electron accumulation, which can facilitate the photocatalytic ORR process [35]. The negative binding energy shift in the C1s XPS spectrum of TPy‐acr COF under light illumination in Figure 4g also demonstrates photo‐generated electron accumulation at C atoms, providing electron‐rich sites for O_2_ activation and reduction [36]. Subsequently, in situ diffuse reflectance infrared Fourier transform spectroscopy (DRIFTS) of TPy‐acr COF under light illumination in a flow of O_2_ was used to capture the possible intermediates involved in this photocatalytic ORR process. As shown in Figure 4h, the bands at 867, 1092, 1370, and 2780 cm^−1^ can be assigned to O−O stretching [37], O_2_∙^−^ vibration [38], O−H vibration [39], and the typical combination mode of O−O−H of the surface adsorbed H_2_O_2_ [40], respectively. As a control experiment, in situ FTIR measurements were also conducted in N_2_ flow (Figure S12). The absence of the characteristic bands with increasing intensity attributed to O_2_∙^−^, O−H, and O−O−H suggests no generation of O_2_∙^−^ and H_2_O_2_. The presence of O_2_∙^−^ was further confirmed by the in situ electron spin resonance (ESR) spectra under light illumination (Figure 4i). The in situ ESR spectra show that TPy‐acr COF exhibits a stronger ESR intensity of trapped O_2_∙^−^ compared to the other two COFs (Figure S13), indicating a higher concentration of unpaired spin electrons and stronger photogenerated electron capability in the TPy‐acr COF photocatalyst [13, 41]. The signal of singlet ^1^O_2_^−^ was also detected by in situ ESR under light illumination; however, the signal dramatically decreased with the addition of benzoquinone (BQ), a scavenger for O_2_∙^−^ (Figure S14). It suggests that the singlet ^1^O_2_^−^ may be generated from the interaction between O_2_∙^−^ and photo‐induced holes via the charge transfer pathway [18, 42]. The 2e^−^ ORR pathway can thus go through O_2_∙^−^ radicals and singlet ^1^O_2_^−^ intermediates toward H_2_O_2_ production [43].

Radical quenching experiments were also conducted to check the participation of other reactive species during the photocatalytic production of H_2_O_2_. Specifically, *tert*‐butanol (TBA), NaIO_3_, and EDTA‐2Na were used as scavengers for ·OH radicals, electrons, and holes, respectively (Figure S15). With increasing amount of TBA, it shows an unobvious quenching effect of TBA for ·OH. To double‐check the existence of ·OH, we further performed the in situ DMPO spin‐trapping ESR tests in H_2_O under N_2_ or O_2_ flow with higher sensitivity (Figure S16). A negligible signal of ·OH was observed in the tests with N_2_, which means that no ·OH intermediate was generated during the WOR process or that any generated ·OH was too strongly adsorbed to desorb from the catalyst surface [43, 44]. In addition, the absence of ·OH signals in N_2_ atmosphere also suggests that neither H_2_O_2_ nor O_2_∙^−^, both of which can be decomposed to form ·OH under strong light illumination if present, was generated during the WOR process. This is consistent with the findings as discussed above (Figures S8–S10). Therefore, the obvious signals of ·OH observed in the tests with O_2_ may probably come from the decomposition products of H_2_O_2_ or O_2_∙^−^ generated during the ORR process (Figure S7) [45]. As anticipated, obvious quenching effects were observed for both electrons (drop by ∼20%) and holes (drop by ∼30%). This further verifies that electrons are consumed for H_2_O_2_ production through the ORR process, which can also be generated during the 4e^−^ WOR process and used for 2e^−^ ORR. Although H_2_O_2_ is not produced through 2e^−^ WOR at holes, the quenching effect of holes suggests that the inhibited WOR limits the ORR process toward H_2_O_2_ production. It is reasonable that the holes are needed for triggering WOR to generate electrons, which participate in ORR to fulfill the entire reaction.

**FIGURE 5:**
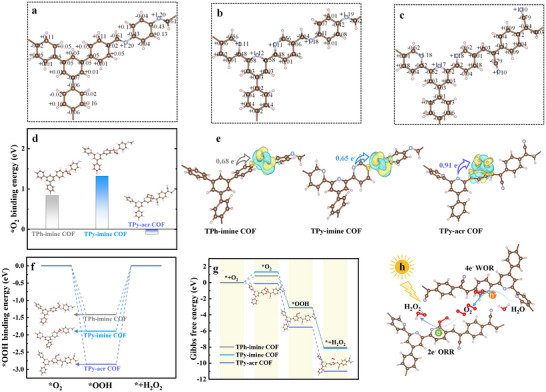
Reaction processes and pathways. (a–c) Bader charge distribution of TPh‐imine COF (a), TPy‐imine COF (b), and TPy‐acr COF (c). (d) Optimized ^*^O_2_ adsorption configurations and the corresponding ^*^O_2_ binding energy of TPh‐imine COF, TPy‐imine COF, and TPy‐acr COF. (e) Charge transfer to O_2_ adsorbed on the optimized adsorption sites of TPh‐imine COF, TPy‐imine COF, and TPy‐acr COF (Yellow and cyan represent electron accumulation and electron depletion, respectively). (f) ^*^OOH binding energy on the optimized adsorption sites of TPh‐imine COF, TPy‐imine COF, and TPy‐acr COF. (g) Gibbs free energy diagrams for ORR toward H_2_O_2_ production over TPh‐imine COF, TPy‐imine COF, and TPy‐acr COF. (h) Schematic illustration of the entire photocatalytic reaction on TPy‐acr COF including 2e^−^ ORR and 4e^−^ WOR.

To gain further insights into the reaction processes and pathways of the two‐electron‐transfer photocatalytic ORR, density functional theory (DFT) calculations were conducted. The adsorption and activation of O_2_ were studied as the initial step of this process. As O_2_ receives photogenerated electrons from the COFs for reduction, the atoms with relatively high electron density tend to serve as active sites. Based on the detailed charge distribution for TPh‐imine COF, TPy‐imine COF, and TPy‐acr COF, as shown in Figure 5a–c, the carbon atoms with high electron density were further investigated as potential adsorption sites for O_2_, in accordance with the in situ XPS results (Figure 4g). As anticipated, TPy‐acr COF with higher performance has much stronger binding of O_2_ than the other two COFs (Figure 5d). However, the other two COFs possess only one effective site for the adsorption of O_2_ (Figures S17–S19). According to the adsorption configuration and differential charge analysis (Figure 5e), obvious charge accumulation at O atoms and charge consumption near the O─O bond suggest the effective activation of the O_2_ on the active site. The adsorbed ^*^O_2_ also gained more charge transfer from TPy‐acr COF compared to the other two COFs, further indicating the enhanced binding and activation of O_2_ as well as faster reaction kinetics over TPy‐acr COF.

The C atom with the lowest energy of O_2_ adsorption was selected as the optimized site for the subsequent calculations. Interestingly, different from the reported study [46], the C atom neighboring the imino N in the terpyridine unit in our work serves as an active site for ORR rather than WOR. As similarly reported, the electron affinity usually determines the types of ORR and WOR on active catalytic centers [25]. ORR is more likely to happen on the C atom neighboring the imino N with more accumulated photo‐generated electrons within TPy‐acr COF (Figures 4g and 5c). The photocatalytic 2e^−^ ORR pathway toward H_2_O_2_ production was further simulated through the stepwise single‐electron ORR route involving the formation of O_2_∙^−^, in consistency with the presence of O_2_∙^−^ in the in situ ESR spectra (Figure 4i) [7]. The binding energy profile results reveal that the adsorption of ^*^OOH is also more stable on TPy‐acr COF (Figure 5f). Furthermore, TPy‐acr COF achieves the lowest energy at all steps in the entire pathway as shown in Figure 5g, suggesting the most favorable thermodynamics toward H_2_O_2_ production on TPy‐acr COF (Figures S20–S22 for all optimized adsorbed configurations).

DFT calculations were also employed to investigate 4e^−^ WOR at holes to fully understand the entire photocatalytic reaction system. As shown in the calculated WOR pathways (Figures S23–S26), both TPh‐imine COF and TPy‐imine COF show three uphill steps from the adsorption of ^*^H_2_O until the formation of ^*^OOH, while TPy‐acr COF shows a downhill step at the adsorption of ^*^OOH, making its WOR pathway toward O_2_ generation more favorable than the other two COFs. The potential oxidation sites are the imino nitrogen atoms in the terpyridine unit of TPy‐acr COF according to our calculation (Figure S27). Consistent with the calculation results, the binding energy of imino N in the N1s XPS spectrum shifted to a higher binding energy position under light illumination (Figure S28), suggesting their electron deficiency under light illumination. Therefore, the holes are localized on the imino N in the terpyridine unit, enabling the 4*e* ^−^ WOR pathway to O_2_ formation (2H_2_O → O_2_ + 4H^+^ + 4*e* ^−^). The photo‐generated electrons are accumulated on the C atom neighboring the imino N in the terpyridine unit, where the 2e^−^ ORR process occurs (O_2_ + 2H^+^ +2*e* ^−^ → H_2_O_2_), completing the entire photocatalytic cycle on the TPy‐acr COF catalyst (2H_2_O + O_2_ → 2H_2_O_2_) (Figure 5h).

## 3 Conclusion

In summary, we have studied a series of covalent organic frameworks by molecular engineering for high‐efficiency photosynthesis of H_2_O_2_. The tailored terpyridine‐based acrylonitrile‐linked COF (TPy‐acr COF) shows asymmetric localized electron distribution near the imine bonds with facilitated charge transfer within the framework. The modulated local electron distribution of the high electron‐negativity carbon atoms adjacent to imine bonds favors the adsorption and activation of ^*^O_2_ on TPy‐acr COF and optimizes the Gibbs free energy at all steps during the pathway toward H_2_O_2_ production. Consequently, the TPy‐acr COF achieved a high H_2_O_2_ production rate of 4.85 mmol g^−1^ h^−1^ from pure water and air without any sacrificial agents and an apparent quantum yield of 2.6%–2.4% at 400–450 nm. This work provides insights into modulating electron distribution asymmetry on COFs through molecular engineering to boost photocatalytic production of H_2_O_2_, guiding rational design of future photocatalysts.

## 4 Experimental Section/Methods

### 4.1 Synthesis of COF samples

#### 4.1.1 Synthesis of TPh‐imine COF

A Pyrex tube with the size of 10 × 8 mm^2^ (o.d × i.d) was filled with benzene‐1,4‐diamine (38.8 mg, 0.36 mmol, 3 equiv) and 5′,5′′′′‐(1,4‐phenylene)bis(([1,1′:3′,1″‐terphenyl]‐4,4″‐dicarbaldehyde)) (72 mg, 0.12 mmol, 1 equiv), o‐dichlorobenzene (1.5 mL), n‐butanol (1.5 mL) and aqueous acetic acid (300 µL, 6 M) sequentially. The mixture was dissolved through ultrasonic. The solution in the glass tube was quickly frozen at 77 K (liquid N_2_ bath), evacuated to an internal pressure of 30 par, and the tube was sealed by flame to keep a length of 16 cm. After natural warm‐up to room temperature, the tube was placed in an oven and heated at 120 °C. Yellow solid was obtained after 7 days and was then isolated by filtration and sequentially refluxed with chloroform and acetone in a Soxhlet extractor for 24 h in each solvent. The material was then activated with supercritical carbon dioxide followed by drying under dynamic vacuum at room temperature for 4 h. The temperature was raised to 120 °C and kept for 2 h to completely activate this COF sample.

#### 4.1.2 Synthesis of TPy‐imine COF

A Pyrex tube with the size of 10 × 8 mm^2^ (o.d × i.d) was filled with benzene‐1,4‐diamine (38.8 mg, 0.36 mmol, 3 equiv.) and 4,4′,4″‐(1,4‐phenylene)bis(([2,2′:6′,2″‐terpyridine]‐5,5″‐dicarbaldehyde)) (78 mg, 0.12 mmol, 1 equiv.), o‐dichlorobenzene (1.5 mL), n‐butanol (1.5 mL), and aqueous acetic acid (300 µL, 6 M) sequentially. The mixture was dissolved through ultrasonic. The solution in the glass tube was quickly frozen at 77 K (liquid N_2_ bath), evacuated to an internal pressure of 30 par, and the tube was sealed by flame to keep a length of 16 cm. After natural warm up to room temperature, the tube was placed in an oven and heated at 120 °C. A yellow solid was obtained after 7 days and was then isolated by filtration and sequentially refluxed with chloroform and acetone in a Soxhlet extractor for 24 h in each solvent. The material was then activated with supercritical carbon dioxide followed by drying under dynamic vacuum at room temperature for 4 h. The temperature was raised to 120 °C and kept for 2 h to completely activate this COF sample.

#### 4.1.3 Synthesis of TPy‐acr COF

A Pyrex tube was charged with p‐phenylenediamine (PDA), 2,2’‐(1,4‐phenylene)‐diacetonitrile (PDAN, 39 mg, 0.25 mmol), and 4′,4′′′′‐(1,4‐phenylene)bis(([2,2′:6′,2″‐terpyridine]‐5,5″‐dicarbaldehyde)) (Tpy, 78.5 mg, 0.12 mmol). To this mixture, o‐dichlorobenzene (1.5 mL) and n‐butanol (1.5 mL) were added, followed by the addition of 600 µL of 1,8‐Diazabicyclo[5.4.0]undec‐7‐ene (DBU, 9 M). The resulting mixture was rapidly frozen at 77 K (liquid N_2_ bath), evacuated to an internal pressure of 30 mTorr, and sealed by flame to maintain a length of 20 cm. After a natural warm‐up to room temperature, the tube was transferred to an oven and heated at 120°C for 7 days. The COF samples were obtained by filtration and subsequently washed with chloroform, water, and acetone. The COF samples were further activated by drying at 120°C for 24 h.

### 4.2 Characterization

Powder X‐ray diffraction (PXRD) characterization was carried out on a Smart Lab diffractometer (Rigaku) utilizing filtered Cu Kα radiation (λ = 1.5406 Å). The UV–vis absorption spectra and UV–vis diffuse reflectance spectroscopy (UV‐DRS) of the samples were carried out on a PerkinElmer LAMBDA 750 spectrophotometer (wavelength range of 300–400 nm) and UV2600 UV–vis spectrophotometer (with BaSO_4_ as reference material), respectively. Fourier transform infrared (FTIR) spectra were collected on the Bruker Vertex 70Hyperion 1000. Photoluminescence (PL) and time‐resolved PL (TRPL) spectra were measured on an Edinburgh FLS1000 fluorescence spectrometer with an excitation wavelength of 420 nm. X‐ray photoelectron spectroscopy (XPS) spectra were obtained on an Xi+ X‐ray photoelectron spectrometer (Axis Supra+ (Kratos Analytical Limited)) with a non‐monochromatized Al‐Kα X‐ray source (hν = 1486.6 eV). Transmission electron microscopy (TEM) images were captured on JEM‐F200.

### 4.3 Photoelectrochemical Measurements

Photoelectrochemical measurements were performed in 0.5 m Na_2_SO_4_ aqueous solution on a CHI660E electrochemical workstation with a typical three‐electrode system. A 300 W Xe lamp was used as the simulated light source, and Ag/AgCl and Pt electrodes were used as the reference electrode and the counter electrode, respectively. A slurry was made by ultrasonically mixing 4 mg of catalyst dispersed in 0.4 mL of anhydrous ethanol and 4 µL of Nafion solution for 30 min, which was then added dropwise onto FTO glass (2 × 2 cm^2^) as a working electrode. The instantaneous photocurrent densities were determined by light on/off cycling under light irradiation at 0.4 V (vs. Ag/AgCl). Also, the electrochemical impedance spectra (EIS) were recorded in the range of 0.01–10^5^ Hz at the same bias voltage. The Mott‐Schottky plots were acquired at a frequency of 1000 Hz and an amplitude of 10 mV.

### 4.4 Photocatalytic H_2_O_2_ Production

A total of 1 mg of photocatalyst was dispersed in a mixed solution containing 50 mL of pure water. The suspension was dispersed homogeneously by ultrasound. A 300 W Xe lamp (CEAULight CEL‐PF300 T6) was used as a simulated illumination. The light average intensity of 350 mW·cm^−2^ was determined by a PL‐MW2000 photoradiometer. The concentration of H_2_O_2_ was measured by a UV–vis spectrophotometer. For example, 1 mL of liquid sample was taken from the reactor and filtered through a 0.22 µm filter to remove the photocatalyst, then diluted with 1 mL of deionized water and mixed with a pre‐prepared 1 mM Ce(SO_4_)_2_ solution. The UV spectrophotometric Ce_2_(SO_4_)_3_ colorimetry method follows Formula (1), in which yellow Ce^4+^ can be reduced by H_2_O_2_ to form colorless Ce^3+^.

(1) $$2Ce^{4+}+H_{2}O_{2}\to 2Ce^{3+}+2H^{+}+O_{2\;}$$

The concentration of H_2_O_2_ can be calculated from Equation (2):

(2) $$c\left(H_{2}O_{2}\right)=1/2\times c\left(Ce^{4+}\right)$$

To obtain the calibration curve, a known concentration of H_2_O_2_ was added to Ce(SO_4_)_2_ solution, and the change in absorption intensity was measured by a UV–vis spectrometer. Based on the linear relationship between Ce^4+^ concentration and signal intensity, the H_2_O_2_ concentration of the photocatalyst can be calculated according to the calibration curve (Figure S29).

More than 30 mg COFs were dispersed in 50 mL deionized water, followed by 30 min sonication. A 300 W Xe lamp was used as simulated lighting. After the reaction, the catalyst was rinsed, filtered, and dried for continuous catalysis (2 h each cycle; 15% of the catalyst was recovered each time). After each recovery, 1 mg of the catalyst was dispersed in 50 mL of deionized water, stirred overnight, and then tested for photocatalytic H_2_O_2_ production. Among them, the photocatalytic process remains consistent.

During the outdoor experiment for H_2_O_2_ photocatalytic production, 75 mg of TPy‐acr COF catalyst was dispersed in 10 mL of pure water for 30 min sonication. The well‐dispersed catalyst ink was added to 1.5 L of pure water, followed by stirring for 1 min. Three independent measurements were conducted every hour to quantify the produced H_2_O_2_. After each hour, the dispersion was stirred for 1 min. The intensity of natural light was measured by a PL‐MW2000 photoradiometer (Figure S30).

The apparent quantum yields of the photocatalysts were determined under a 300 W Xenon lamp. The active area of the photocatalytic reactor is about 15.9 cm^2^. Monochromatic light intensities were measured at four representative points using a PL‐MW2000 photoradiometer. Then, the AQY was calculated using Equation (3) as follows:

(3) $$\mathrm{AQY}\;=\frac{2\;\times \;h\;\times \;n\;\times \;N_{A}\;\times \;c}{S\;\times \;P\;\times \;t\;\times \;\lambda }\;\times 100\%$$

*n*, the amount of H_2_O_2_ molecules (mol); h, Planck constant (6.626 × 10^−34^ Js); N_A_, Avogadro constant (6.022 × 10^23^ mol^−1^); c, the speed of light (3 × 10^8^ m s^−1^); S, the active area of the photocatalytic reactor (cm^2^); P, irradiation intensity (W·cm^−2^); t, photoreaction time (s); λ, filter wavelength (m).

### 4.5 Mechanistic Investigation

TPy‐acr COF (2 mg) and H_2_^18^O (0.5 mL) were added to a glass vial (2 mL). The suspension was well dispersed by sonication for 5 min and bubbled with air for 15 min in the dark, before sealing off with a rubber septum. A 300W Xenon lamp was used to irradiate the reaction mixture with constant coil fan cooling for 12 h. A total of 100 µL gas in the headspace was injected for analysis by GC‐MS.

In situ diffuse reflectance infrared Fourier transform spectroscopy (DRIFTS) measurements were performed on a Thermo Fisher Scientific ESCALAB 250Xi. The photocatalyst sample was loaded into an in situ infrared holder in the chamber. First, the chamber was degassed under argon flow at a rate of 20 mL min^−1^ for 30 min. Oxygen and water vapor were then purged into the chamber for 30 min in the dark. The in situ DRIFTS spectra were collected at specific intervals under visible light (λ = 420–600 nm) through the window of the chamber.

In situ Kelvin probe force microscopy (KPFM) experiments were conducted on a Bruker Dimension Icon. In situ XPS measurements were performed on a Bruker INVENIO‐S X‐ray photoelectron spectrometer. In situ ESR spectra were recorded on a Bruker‐Magnettech ESR 5000 at room temperature. A 300W Xe lamp was used for light illumination for these in situ characterizations. For the in situ ESR tests of ∙O_2_^−^, 5 mg of photocatalyst was dispersed into the photocatalytic reaction cell containing methanol (10 mL), followed by bubbling O_2_ for 10 min before the tests. For the in situ ESR tests of ∙OH, 5 mg of photocatalyst was dispersed into the photocatalytic reaction cell containing water (10 mL), followed by bubbling N_2_ or O_2_ for 10 min before the tests. A Xe lamp was used as the light source to irradiate the reaction cell during tests.

Rotating ring‐disk electrode (RRDE) measurements were carried out on an ALS RRDE‐3A equipment in a three‐electrode system including an RRDE as the working electrode, a graphite rod as the counter electrode, and an Ag/AgCl electrode as the reference electrode. The RRDE electrode consists of a glassy carbon disk (disk diameter = 4 mm) and a platinum ring (ring size ID/OD = 5 mm/7 mm). COF samples (5 mg) were dispersed in a 1 mL mixture of EtOH and H_2_O (1:1) containing Nafion (50 µL), followed by ultrasonication for 30 min. 20 µL of ink was drop‐cast on the disk electrode and dried at room temperature. N_2_ gas was purged into the electrolyte for 30 min before measurements. The linear sweep voltammetry (LSV) curves were recorded in a 0.1 M phosphate buffer solution (pH = 7) at a scan rate of 10 mV s^−1^ and a rotation rate of 1600 rpm. The potential of the ring electrode was set to −0.23 and 0.6 V (vs. Ag/AgCl) to detect O_2_ or H_2_O_2_, respectively [26].

### 4.6 Computational Methods

All spin‐polarized density functional theory (DFT) calculations were performed by the Vienna Ab initio Simulation Package (VASP) [47] based on the projector‐augmented wave (PAW) method [48]. The periodic models of the materials were obtained using full structural optimization, including the unit cell shape and volume. Higher precision optimizations for selected structures as well as all electronic property calculations were then performed using DFT calculations using the PAW method and Perdew−Burke−Ernzerhof exchange–correlation functional [49], with Grimme's D3 approach [50] to the London dispersion correction. During structure optimization and static calculations, we employed a cut‐off energy of 400 eV for the plane‐wave basis set and set 0.01 eV Å^−1^ as the force convergence criterion. In the self‐consistent field iteration, we used the energy convergence criterion of 10^−5^ eV for the optimization and the static calculation, respectively. We used a k‐mesh (which is a set of points in the Brillouin zone used to sample the electronic structure of a material) of 1 × 1 × 1 for the structural relaxation and the converged charge density.

To simulate the 2e^−^ oxygen reduction reaction (ORR) toward H_2_O_2_ generation, we constructed a 1 × 1 × 1 single cell of the COFs. We applied a computational hydrogen electrode model to derive the Gibbs free energies with Nørskov's approach [51]. Each reaction step is simulated in an orthorhombic cell with in‐plane lattice constants of a = 37.0 Å and b = 38.0 Å, with a 15 Å vacuum layer to prevent periodic images between adjacent layers. The Gibbs free energy for each step was calculated with the following Equation (4):

(4) $$\Delta G=\Delta E_{DFT}+\Delta E_{ZPE}-T\Delta S$$

where Δ*E_DFT_* is the total energy of a certain optimized structure with reaction intermediates from the DFT simulation. Δ*E_ZPE_* is the zero‐point energy calculated from vibrational frequencies, *T* is the temperature (298.15 K), *S* is the entropy obtained from standard thermodynamics tables [52] or from vibrational frequencies. In this work, Δ*E_ZPE_* and *S* were directly obtained from frequency calculations using VASPKIT [53].

To investigate the 4e^−^ water oxidation reaction (WOR) pathway, the same computational setup, convergence criteria, and theoretical framework described above were strictly adopted. The WOR mechanism follows the standard associative pathway, involving the sequential proton‐coupled electron transfer (PCET) steps: ^*^H_2_O → ^*^OH → ^*^O → ^*^OOH → O_2_ (g). All slab models, vacuum spacing (15 Å), DFT functional (PBE+D3), plane‐wave cutoff (400 eV), k‐point sampling, and post‐processing tools remain fully consistent with those employed for the ORR calculations.

Work functions were calculated by subtracting the vacuum potential of the simulation cell from the Fermi energy of the same simulation cell. Vacuum electronic binding energy for ^*^OOH was calculated according to the following Equation (5),

(5) $$\Delta \;E_{*OOH}=E_{slab+*OOH}\;-E_{slab}-E_{OOH}$$

where E_slab+*OOH_ and *E_slab_* are the total energies of the slab with and without the adsorbed ^*^OOH. *E_OOH_* is the total energy of the ^*^OOH intermediate.

For structural relaxation, a 1 × 1 × 1 Gamma‐centered k‐mesh was used, and a 1 × 1 × 1 Gamma‐centered k‐mesh was employed for Bader charge analysis. Bader charge analysis is used to construct the charge distribution map of catalysts [54]. Charge density difference analysis was generated using VESTA [55]. The distribution of the highest occupied molecular orbital (HOMO) and the lowest unoccupied molecular orbital (LUMO) was calculated using hybrid density functional theory as implemented in VASP. Specifically, the PBE0 functional was employed in conjunction with the PAW method [56]. The orbital occupation numbers for HOMO and LUMO were 2 and 0, respectively.

## Funding

This work was supported by the National Natural Science Foundation of China Young Scientists Fund (22408298), the National Natural Science Foundation of China (52472257), and CNPC Innovation Foundation (No. 2024DQ02‐0311) resources.

## Conflicts of Interest

The authors declare no conflict of interest.

## Supporting information




**Supporting File**: adma73821‐sup‐0001‐SuppMat.docx.

## Data Availability

The data that support the findings of this study are available from the corresponding author upon reasonable request

## References

[1] A. G. Fink , R. S. Delima , A. R. Rousseau , et al., “Indirect H_2_O_2_ Synthesis Without H_2_ ,” Nature Communications 15, no. 1 (2024): 766. 10.1038/s41467-024-44741-1 PMC10817937 38278793

[2] A. T. Murray , S. Voskian , M. Schreier , T. A. Hatton , and Y. Surendranath , “Electrosynthesis of Hydrogen Peroxide by Phase‐Transfer Catalysis,” Joule 3, no. 12 (2019): 2942.

[3] Hydrogen Peroxide (HP): 2026 World Market Outlook and Forecast up to 2035 (Merchant Research & Consulting, Ltd, 2026), https://mcgroup.co.uk/researches/hydrogen‐peroxide‐hp.

[4] Hydrogen Peroxide Market Size & Share Analysis – Growth Trends and Forecast (2026‐2031) (Mordor Intelligence, 2026), https://www.mordorintelligence.com/industry‐reports/hydrogen‐peroxide‐market.

[5] C. Kormann , D. W. Bahnemann , and M. R. Hoffmann , “Photocatalytic Production of Hydrogen Peroxides and Organic Peroxides in Aqueous Suspensions of Titanium Dioxide, Zinc Oxide, and Desert Sand,” Environmental Science & Technology 22, no. 7 (1988): 798–806. 22195664 10.1021/es00172a009

[6] T. Liu , Z. Pan , J. J. M. Vequizo , et al., “Overall Photosynthesis of H_2_O_2_ by an Inorganic Semiconductor,” Nature Communications 13, no. 1 (2022): 1034. 10.1038/s41467-022-28686-x PMC8873311 35210427

[7] L. Wang , J. Zhang , Y. Zhang , H. Yu , Y. Qu , and J. Yu , “Inorganic Metal‐Oxide Photocatalyst for H_2_O_2_ Production,” Small 18, no. 8 (2022): 2104561. 10.1002/smll.202104561 34716646

[8] H. Ling , H. Sun , L. Lu , et al., “Sustainable Photocatalytic Hydrogen Peroxide Production over Octonary High‐Entropy Oxide,” Nature Communications 15, no. 1 (2024): 9505. 10.1038/s41467-024-53896-w PMC11532407 39489764

[9] S. Deng , W.‐P. Xiong , G.‐X. Zhang , et al., “Metal‐Free Modification Overcomes the Photocatalytic Limitations of Graphitic Carbon Nitride: Efficient Production and In Situ Application of Hydrogen Peroxide,” Advanced Energy Materials 14, no. 39 (2024): 2401768.

[10] H. Li , S. Kelly , D. Guevarra , et al., “Analysis of the Limitations in the Oxygen Reduction Activity of Transition Metal Oxide Surfaces,” Nature Catalysis 4, no. 6 (2021): 463–468.

[11] Y. Li , Y. Zhao , J. Wu , et al., “Photo‐Charge Regulation of Metal‐Free Photocatalyst by Carbon Dots for Efficient and Stable Hydrogen Peroxide Production,” Journal of Materials Chemistry A 9, no. 45 (2021): 25453–25462.

[12] Y. Chen and D. Jiang , “Photocatalysis with Covalent Organic Frameworks,” Accounts of Chemical Research 57, no. 21 (2024): 3182–3193. 39370855 10.1021/acs.accounts.4c00517

[13] Y. Liu , L. Li , Z. Sang , et al., “Enhanced Hydrogen Peroxide Photosynthesis in Covalent Organic Frameworks through Induced Asymmetric Electron Distribution,” Nature Synthesis 4 (2024): 134–141.

[14] D. Yu , L. Xu , K. Fu , et al., “Electronic Structure Modulation of Iron Sites with Fluorine Coordination Enables Ultra‐Effective H_2_O_2_ Activation,” Nature Communications 15, no. 1 (2024): 2241. 10.1038/s41467-024-46653-6 PMC10933296 38472214

[15] L. Zhang , S.‐C. Wan , J. Zhang , et al., “Activation of Pyroptosis Using Aiegen‐Based sp^2^ Carbon‐Linked Covalent Organic Frameworks,” Journal of the American Chemical Society 145, no. 32 (2023): 17689–17699. 37550880 10.1021/jacs.3c04027

[16] L. Zhang , S. Wang , Y. Zhou , C. Wang , X.‐Z. Zhang , and H. Deng , “Covalent Organic Frameworks as Favorable Constructs for Photodynamic Therapy,” Angewandte Chemie International Edition 58, no. 40 (2019): 14213–14218. 31347259 10.1002/anie.201909020

[17] Y. Chen , R. Liu , Y. Guo , et al., “Hierarchical Assembly Of Donor–Acceptor Covalent Organic Frameworks For Photosynthesis Of Hydrogen Peroxide From Water And Air,” Nature Synthesis 3, no. 8 (2024): 998–1010.

[18] J.‐Y. Yue , J.‐X. Luo , Z.‐X. Pan , Q. Xu , P. Yang , and B. Tang , “Phenanthridine‐based Covalent Organic Frameworks for Boosting Overall Solar H_2_O_2_ Production,” Angewandte Chemie International Edition 64, no. 5 (2025): 202417115. 10.1002/anie.202417115 39363753

[19] R. Sun , X. Yang , X. Hu , et al., “Unprecedented Photocatalytic Hydrogen Peroxide Production via Covalent Triazine Frameworks Constructed from Fused Building Blocks,” Angewandte Chemie International Edition 64, no. 4 (2025): 202416350. 10.1002/anie.202416350 39247985

[20] R. Liu , Y. Chen , H. Yu , et al., “Linkage‐Engineered Donor–Acceptor Covalent Organic Frameworks For Optimal Photosynthesis Of Hydrogen Peroxide From Water And Air,” Nature Catalysis 7, no. 2 (2024): 195–206.

[21] J.‐Y. Yue , J.‐X. Luo , Z.‐X. Pan , et al., “Regulating the Topology of Covalent Organic Frameworks for Boosting Overall H_2_O_2_ Photogeneration,” Angewandte Chemie International Edition 63, no. 24 (2024): 202405763. 10.1002/anie.202405763 38607321

[22] L. Zhang , C. Wang , Q. Jiang , P. Lyu , and Y. Xu , “Structurally Locked High‐Crystalline Covalent Triazine Frameworks Enable Remarkable Overall Photosynthesis of Hydrogen Peroxide,” Journal of the American Chemical Society 146, no. 43 (2024): 29943–29954. 39418115 10.1021/jacs.4c12339

[23] Q. Liao , Q. Sun , H. Xu , et al., “Regulating Relative Nitrogen Locations of Diazine Functionalized Covalent Organic Frameworks for Overall H_2_O_2_ Photosynthesis,” Angewandte Chemie International Edition 62, no. 41 (2023): 202310556. 10.1002/anie.202310556 37632257

[24] J.‐Y. Yue , L.‐P. Song , Y.‐F. Fan , et al., “Thiophene‐Containing Covalent Organic Frameworks for Overall Photocatalytic H_2_O_2_ Synthesis in Water and Seawater,” Angewandte Chemie International Edition 62, no. 38 (2023): 202309624. 10.1002/anie.202309624 37526096

[25] A. Chakraborty , A. Alam , U. Pal , et al., “Enhancing Photocatalytic Hydrogen Peroxide Generation by Tuning Hydrazone Linkage Density in Covalent Organic Frameworks,” Nature Communications 16, no. 1 (2025): 503. 10.1038/s41467-025-55894-y PMC11711387 39779748

[26] C. Qin , X. Wu , L. Tang , et al., “Dual Donor‐Acceptor Covalent Organic Frameworks for Hydrogen Peroxide Photosynthesis,” Nature Communications 14, no. 1 (2023): 5238. 10.1038/s41467-023-40991-7 PMC10462664 37640726

[27] R.‐M. Zhu , Y. Liu , W.‐K. Han , et al., “Three‐Dimensional Covalent Organic Frameworks Based on Linear and Trigonal Linkers for High‐Performance H_2_O_2_ Photosynthesis,” Angewandte Chemie International Edition 64, no. 1 (2025): 202412890. 10.1002/anie.202412890 39148428

[28] W. Zhao , P. Yan , B. Li , et al., “Accelerated Synthesis and Discovery of Covalent Organic Framework Photocatalysts for Hydrogen Peroxide Production,” Journal of the American Chemical Society 144, no. 22 (2022): 9902–9909. 35635501 10.1021/jacs.2c02666 PMC9185744

[29] F. Liu , P. Zhou , Y. Hou , et al., “Covalent Organic Frameworks for Direct Photosynthesis of Hydrogen Peroxide from Water, Air and Sunlight,” Nature Communications 14, no. 1 (2023): 4344. 10.1038/s41467-023-40007-4 PMC10356944 37468482

[30] G. Fu , D. Yang , S. Xu , et al., “Construction of Thiadiazole‐Bridged sp^2^‐Carbon‐Conjugated Covalent Organic Frameworks with Diminished Excitation Binding Energy toward Superior Photocatalysis,” Journal of the American Chemical Society 146, no. 2 (2024): 1318–1325. 38181378 10.1021/jacs.3c08755

[31] T. Kirchartz , J. A. Márquez , M. Stolterfoht , and T. Unold , “Photoluminescence‐Based Characterization of Halide Perovskites for Photovoltaics,” Advanced Energy Materials 10, no. 26 (2020): 1904134.

[32] H. Tu , B. Tian , S. Chen , et al., “Enhancing Photocatalytic Efficiency through Surface Modification to Manipulate Internal Electron‐Hole Distribution,” npj Clean Water 8, no. 1 (2025): 48.

[33] Q. Xue , H. Li , P. Jin , X. Zhou , and F. Wang , “Singlet‐Oxygen‐Driven Cooperative Photocatalytic Coupling of Biomass Valorization and Hydrogen Peroxide Production Using Covalent Organic Frameworks,” Angewandte Chemie International Edition 64, no. 19 (2025): 202423368. 10.1002/anie.202423368 40035701

[34] Q. Liu , H. Bi , R. Zhao , X. Yang , F. Chen , and Z. Shen , “Fully Exposed Silver Clusters Enabling Highly Efficient Photocatalytic H_2_O_2_ Production in Pure Water,” Angewandte Chemie International Edition 64, no. 37 (2025): 202511687. 10.1002/anie.202511687 40685675

[35] F. Chen , G. Cao , Q. Liu , Y. Duan , W. Li , and Z. Shen , “Bi─O Bridges Trigger Lattice Strain‐Electronic Synergy at Inherent In Sites in ZnIn_2_S_4_ for Boosting Solar‐to‐H_2_O_2_ Conversion,” Angewandte Chemie International Edition 64, no. 47 (2025): 202518232. 10.1002/anie.202518232 40985709

[36] M. Xu , X. Ruan , X. Zhang , et al., “Localized Oxygen Enrichment in a Covalent Organic Framework–ZnIn_2_S_4_S‐Scheme Heterojunction Enables Spatially Confined Oxygen Reduction and Boosts Photocatalytic H_2_O_2_ Selectivity,” Nano Letters 25, no. 35 (2025): 13315–13325. 40838873 10.1021/acs.nanolett.5c03476

[37] H. F. Shurvell and M. C. Southby , “Infrared and Raman Spectra of Tetrahydrofuran Hydroperoxide,” Vibrational Spectroscopy 15, no. 1 (1997): 137–146.

[38] V. Briega‐Martos , W. Cheuquepán , and J. M. Feliu , “Detection of Superoxide Anion Oxygen Reduction Reaction Intermediate on Pt(111) by Infrared Reflection Absorption Spectroscopy in Neutral Ph Conditions,” The Journal of Physical Chemistry Letters 12, no. 6 (2021): 1588–1592. 33539102 10.1021/acs.jpclett.0c03510 PMC8460065

[39] W. W. Simons , ed., The Sadtler Handbook of Infrared Spectra (Sadtler Research Laboratories, 1978).

[40] A. V. Churakov , S. Sladkevich , O. Lev , T. A. Tripol'skaya , and P. V. Prikhodchenko , “Cesium Hydroperoxostannate: First Complete Structural Characterization of a Homoleptic Hydroperoxocomplex,” Inorganic Chemistry 49, no. 11 (2010): 4762–4764. 20459061 10.1021/ic100554u

[41] H. Tan , P. Zhou , M. Liu , et al., “Photocatalysis of Water into Hydrogen Peroxide Over an Atomic Ga‐N_5_ Site,” Nature Synthesis 2, no. 6 (2023): 557–563.

[42] J. Yang , X. Zeng , B. Zhu , et al., “Self‐Trapped Excitons Activate Pseudo‐Inert Basal Planes of 2D Organic Semiconductors for Improved Photocatalysis,” Advanced Materials 37, no. 30 (2025): 2505653. 40377363 10.1002/adma.202505653 PMC12306381

[43] X. Sun , J. Yang , X. Zeng , et al., “Pairing Oxygen Reduction and Water Oxidation for Dual‐Pathway H_2_O_2_ Production,” Angewandte Chemie International Edition 63, no. 52 (2024): 202414417. 10.1002/anie.202414417 39308269

[44] Z. Yong and T. Ma , “Solar‐to‐H_2_O_2_ Catalyzed by Covalent Organic Frameworks,” Angewandte Chemie International Edition 62, no. 49 (2023): 202308980. 10.1002/anie.202308980 37574706

[45] J. Yang , X. Zeng , M. Tebyetekerwa , et al., “Engineering 2D Photocatalysts for Solar Hydrogen Peroxide Production,” Advanced Energy Materials 14, no. 23 (2024): 2400740.

[46] J. Zhang , F. Xue , and Z. Wang , “Terpyridine‐ and Quarterpyridine‐Based Cationic Covalent Organic Frameworks for Visible‐Light‐Catalytic H_2_O_2_ Synthesis,” Angewandte Chemie International Edition 64, no. 18 (2025): 202425617. 10.1002/anie.202425617 39963951

[47] G. Kresse and J. Furthmüller , “Efficient Iterative Schemes for ab Initio Total‐Energy Calculations Using a Plane‐Wave Basis Set,” Physical Review B 54, no. 16 (1996): 11169–11186. 10.1103/physrevb.54.11169 9984901

[48] P. E. Blöchl , “Projector Augmented‐Wave Method,” Physical Review B 50, no. 24 (1994): 17953. 10.1103/physrevb.50.17953 9976227

[49] J. P. Perdew , K. Burke , and M. Ernzerhof , “Generalized Gradient Approximation Made Simple,” Physical Review Letters 77, no. 18 (1996): 3865–3868. 10062328 10.1103/PhysRevLett.77.3865

[50] S. Grimme , J. Antony , S. Ehrlich , and H. Krieg , “A Consistent and Accurate ab Initio Parametrization of Density Functional Dispersion Correction (DFT‐D) for the 94 Elements H‐Pu,” The Journal of Chemical Physics 132, no. 15 (2010): 154104. 20423165 10.1063/1.3382344

[51] J. K. Nørskov , T. Bligaard , A. Logadottir , et al., “Trends in the Exchange Current for Hydrogen Evolution,” Journal of the Electrochemical Society 152, no. 3 (2005): J23.

[52] J. K. Nørskov , J. Rossmeisl , A. Logadottir , et al., “Origin of the Overpotential for Oxygen Reduction at a Fuel‐Cell Cathode,” The Journal of Physical Chemistry B 108, no. 46 (2004): 17886. 39682080 10.1021/jp047349j

[53] V. Wang , N. Xu , J.‐C. Liu , G. Tang , and W.‐T. Geng , “Vaspkit: A User‐Friendly Interface Facilitating High‐Throughput Computing and Analysis Using Vasp Code,” Computer Physics Communications 267 (2021): 108033.

[54] G. Henkelman , A. Arnaldsson , and H. Jónsson , “A Fast and Robust Algorithm for Bader Decomposition of Charge Density,” Computational Materials Science 36, no. 3 (2006): 354–360.

[55] K. Momma and F. Izumi , “VESTA 3 for Three‐Dimensional Visualization Of Crystal, Volumetric And Morphology Data,” Journal of Applied Crystallography 44, no. 6 (2011): 1272–1276.

[56] C. Adamo and V. Barone , “Toward Reliable Density Functional Methods without Adjustable Parameters: The PBE0 Model,” The Journal of Chemical Physics 110, no. 13 (1999): 6158–6170.

