# Supplementary material for: Enhanced Photocatalytic H2O2 Production on Terpyridine‐Based Acrylonitrile‐Linked Covalent Organic Frameworks with Asymmetric Localized Electron Distribution
Source: Adv Mater. 2026 Jun 20;38(42):e73821. doi: 10.1002/adma.73821 (PMC13410879; doi:10.1002/adma.73821)
Supplement: Supplementary file 1 — Supporting File: adma73821‐sup‐0001‐SuppMat.docx. [file ADMA-38-e73821-s001.docx]

***Supporting Information***

**Enhanced Photocatalytic H_2_O_2_ Production on Terpyridine-based Acrylonitrile-linked Covalent Organic Frameworks with Asymmetric Localized Electron Distribution**

Qinglan Zhao,^a,+^ Mengmeng Ce,^b,+^ Hengtao Lei,^c,+^ Tianyun Jin,^d^ Zhipeng Xie,^e^ Yushen Liu,^a^ Shengyi Yang,^d^ Xinwen Ou,^d^ Mohammad Farhadpour,^a^ Song-Zhu Kure-Chu,^f^ Xuan-He Liu,^*,b^ Liang Zhang,^*,c^ and Jimmy C. Yu^*,e^

^a^ Department of Chemical and Biological Engineering, The Hong Kong University of Science and Technology, Clear Water Bay, Kowloon, Hong Kong, China

^b^ School of Science, China University of Geosciences (Beijing), Beijing, 100083 China

^c^ Key Laboratory of Precision and Intelligent Chemistry, School of Chemistry and Materials Science, University of Science and Technology of China, Hefei, 230026, China

^d^ Department of Chemistry, The Hong Kong University of Science and Technology, Clear Water Bay, Kowloon, Hong Kong, China

^e^ Department of Chemistry, The Chinese University of Hong Kong, Hong Kong, China

^f^ School of Materials Science and Engineering, Southeast University, Nanjing 211189, China

^+^ These authors contributed equally.

**Figure S1**. Pore size distribution curves of (a) TPh-imine COF, (b) TPy-imine COF and (c) TPy-acr COF.


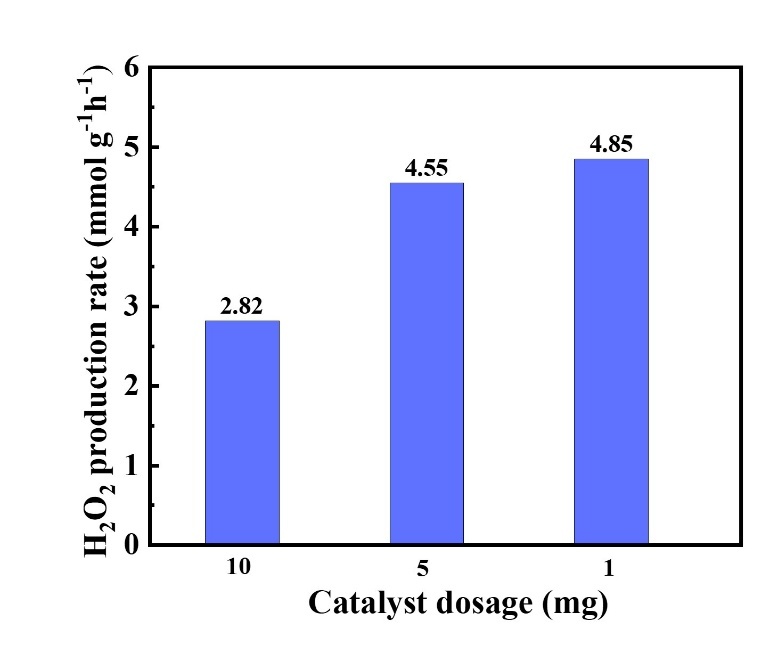


**Figure S2**. Average photocatalytic H_2_O_2_ production rate over TPy-acr COF as a function of catalyst dosage in 50 mL of pure water.

**Figure S3**. Photocatalytic production and rate with time over (a) TPh-imine COF, (b) TPy-imine COF and (c) TPy-acr COF.

**Figure S4**. SEM images of TPy-acr COF (a) before and (b) after photocatalytic reaction.


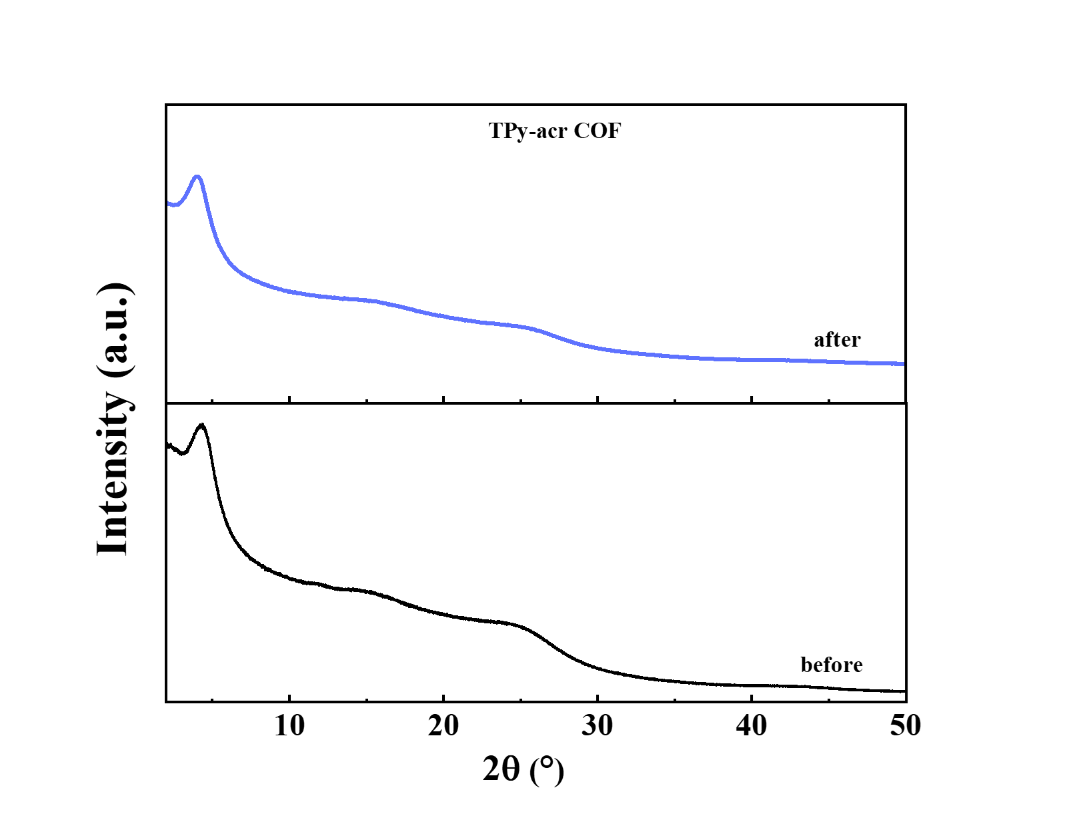


**Figure S5**. XRD patterns of TPy-acr COF before and after photocatalytic reaction.


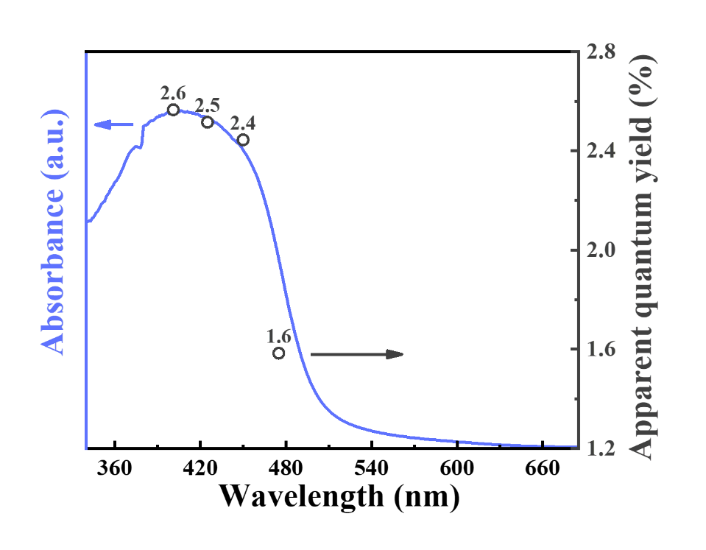


**Figure S6**. Apparent quantum efficiency (black circles) and electronic absorption spectrum (light purple curve) of TPy-acr COF.


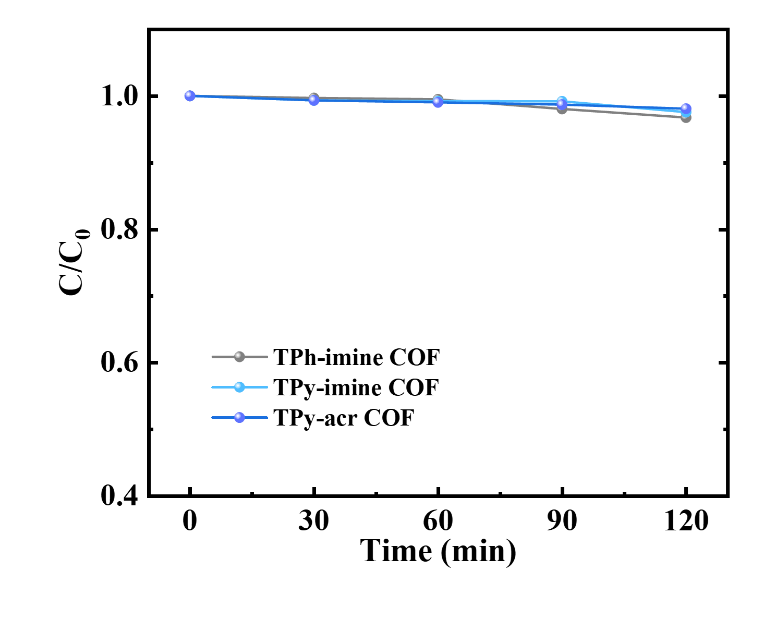


**Figure S7**. H_2_O_2_ decomposition data of TPh-imine COF, TPy-imine COF and TPy-acr COF. C/C_0_ is the ratio of the remaining concentration of H_2_O_2_ to the initial concentration.


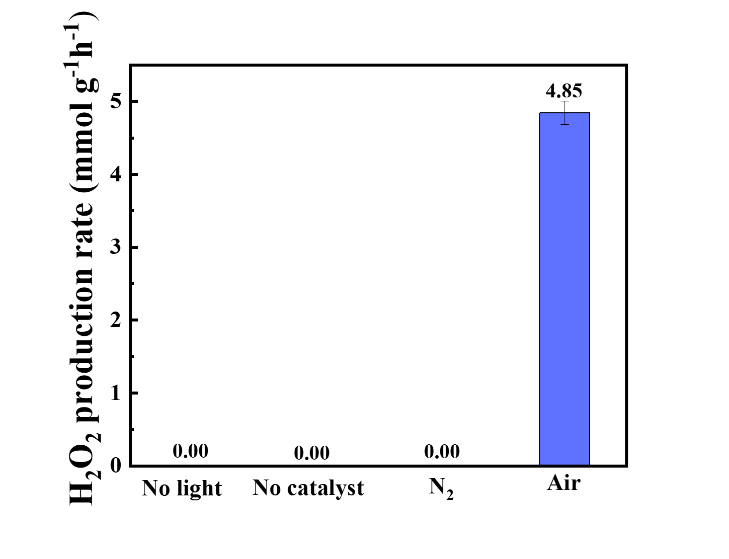


**Figure S8**. H_2_O_2_ production rate over TPy-acr COF under different conditions.

**Figure S9**. GC-MS chromatogram of (a) the air and of (b) the headspace of reaction system with H_2_^18^O.

**Figure S10**. (a) RRDE LSV curves obtained in 0.1 M phosphate buffer solution at a scan rate of 10 mV s^−1^ under rotation of 1600 rpm. The potential of Pt ring electrode is set at 0.6 V *vs*. Ag/AgCl to detect H_2_O_2_. (b) RRDE LSV curves obtained in 0.1 M phosphate buffer solution at a scan rate of 10 mV s^−1^ under rotation of 1600 rpm. The potential of Pt ring electrode is set at −0.23 V *vs*. Ag/AgCl to detect O_2_.

**Figure S11**. (a) Steady-state PL spectra and (b) TRPL spectra of COF samples (circle symbol: experimental result, solid line: fit data).

**Figure S12**. Time-dependent *in situ* DRIFTS spectra of TPy-acr COF under light illumination in N_2_ flow. The absence of the characteristic bands as labeled suggests no generation of O_2_∙^−^ and H_2_O_2_.


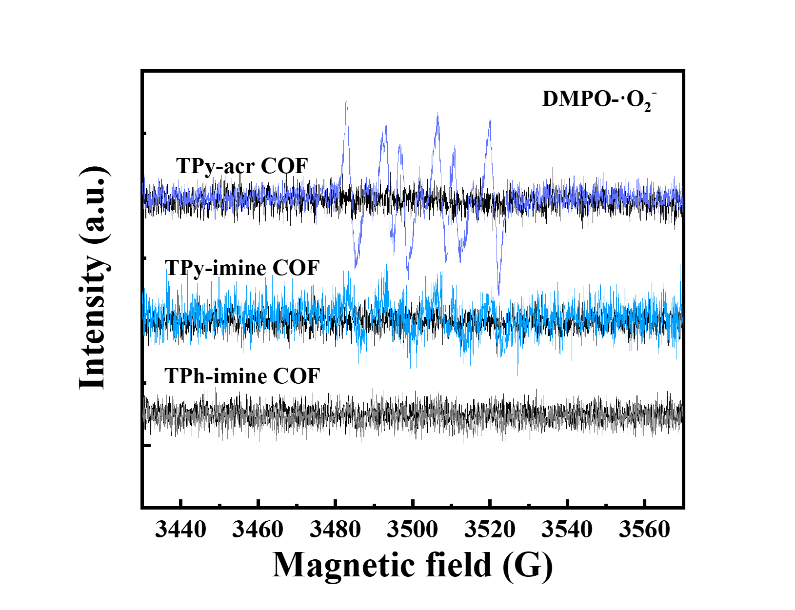


**Figure S13**. DMPO spin-trapping ESR spectra of all COF samples for detecting O_2_∙^−^ in methanol without light illumination (black curves for all corresponding samples) and with light illumination for 15 min.


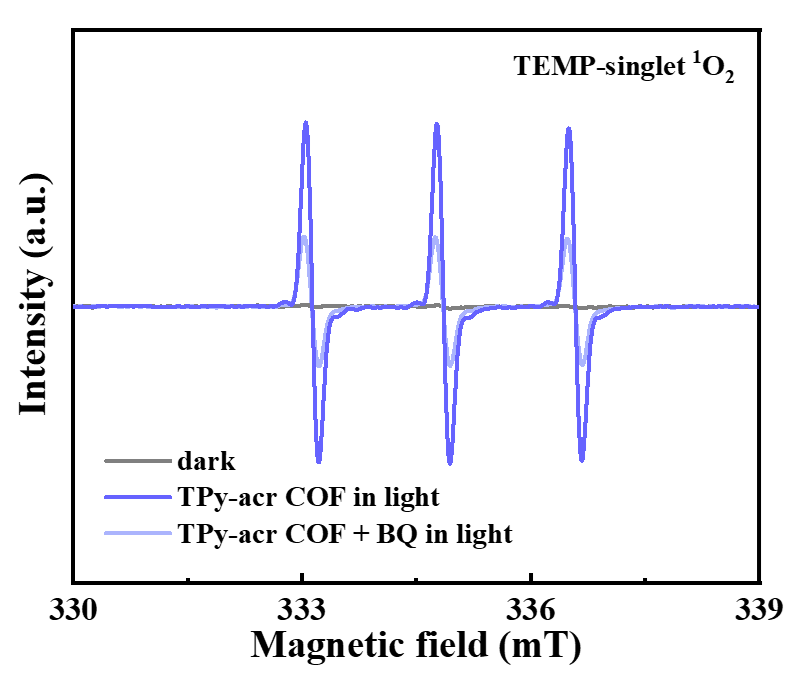


**Figure S14**. Singlet ^1^O_2_ generated ESR signals by TPy-acr COF trapped by TEMP with and without benzoquinone (BQ) in the dark and light for 15 min.

**Figure S15**. The plots of H_2_O_2_ concentration degradation on TPy-acr COF with time using (a) 5 mM or 10 mM tert-butanol (TBA), (b) 0.1 mM NaIO_3_, and (c) 0.1 mM EDTA-2Na as scavengers for ·OH radicals, electrons, and holes, respectively. *C* is the H_2_O_2_ concentration measured at certain time with the addition of a specific scavenger, and *C_0_* is the H_2_O_2_ concentration measured in a control experiment without the corresponding scavenger.

**Figure S16**. DMPO spin-trapping ESR spectra of all COF samples in (a) N_2_ and (b) O_2_ for detecting ∙OH in H_2_O without light illumination (black curves for all corresponding samples) and with light illumination for 15 min.

**Figure S17**. (a) The potential sites for adsorbing O_2_ on TPy-acr COF and (b) the calculated *O_2_ binding energy values with the corresponding adsorption configurations. (C2~C4 sites are successful for *O_2_ adsorption according to the investigation of four potential sites.)

**Figure S18**. (a) The potential sites for adsorbing O_2_ on TPy-imine COF and (b) the optimized *O_2_ adsorption configuration. (C2 is the only site for successful *O_2_ adsorption according to the investigation of four potential sites.)

**Figure S19**. (a) The potential sites for adsorbing O_2_ on TPh-imine COF and (b) the optimized *O_2_ adsorption configuration. (C2 is the only site for successful *O_2_ adsorption according to the investigation of two potential sites.)

**Figure S20**. Schematic of adsorption configurations for H_2_O_2_ formation over TPy-acr COF.

**Figure S21**. Schematic of adsorption configurations for H_2_O_2_ formation over TPy-imine COF.

**Figure S22**. Schematic of adsorption configurations for H_2_O_2_ formation over TPh-imine COF.


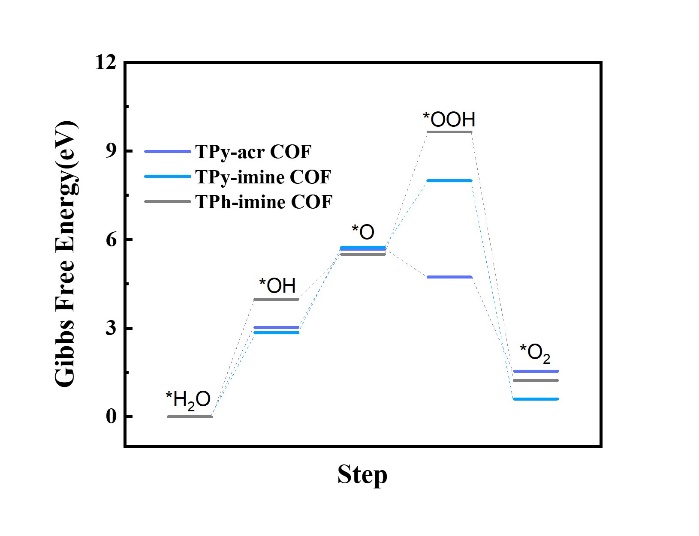


**Figure S23**. The simulated 4*e*^–^ WOR pathways to O_2_ formation on TPh-imine COF, TPy-imine COF and TPy-acr COF.

**Figure S24**. Schematic of optimized adsorption configurations for 4*e*^–^ WOR toward O_2_ formation over TPy-acr COF.

**Figure S25**. Schematic of optimized adsorption configurations for 4*e*^–^ WOR toward O_2_ formation over TPy-imine COF.

**Figure S26**. Schematic of optimized adsorption configurations for 4*e*^–^ WOR toward O_2_ formation over TPh-imine COF.


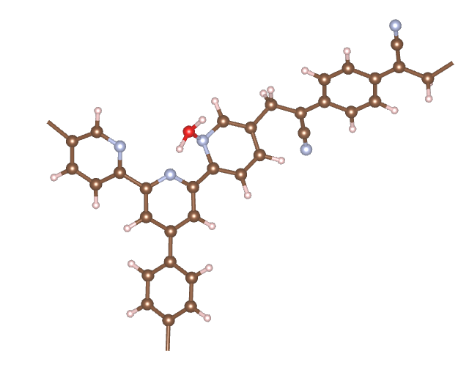


**Figure S27**. Optimized adsorption configuration of *H_2_O on TPy-acr COF.

**Figure S28**. *In situ* N1s XPS spectra of TPy-acr COF with light illumination (a) off and (b) on.

**Figure S29**. (a) UV-vis absorbance of standard samples with various concentrations and (b) the related calibration curve.

**Figure S30**. A photo of the intensity of natural light measured by the PLMW2000 photoradiometer during the outdoor experiment.

**Table S1**. Pore properties of the prepared COF samples in this work.

| Sample | S_BET_ (m^2^ g^-1^) | Pore volume (cm^3^ g^-1^) | Peak pore width (nm) |
| --- | --- | --- | --- |
| TPh-imine COF | 1466.12 | 0.904 | 2.351 |
| TPy-imine COF | 1519.30 | 0.993 | 2.351 |
| TPy-acr COF | 568.79 | 0.395 | 1.848 |

**Table S2**. Comparison of production rates of H_2_O_2_ in pure water and air for TPy-acr COF with recently reported COF photocatalysts.

| Sample | Production rate (mmol g^-1^ h^-1^) | Reference |
| --- | --- | --- |
| TPy-acr COF | 4.85 | This work |
| TP-DPBD3O-COF | 7.2 | ^1^ |
| PD-COF2 | 6.103 | ^2^ |
| CTF-BTT | 5.757 | ^3^ |
| Hz-TPBT-COF | 5.7 | ^4^ |
| TBD-COF | 5.448 | ^5^ |
| CTF-NSs | 5.007 | ^6^ |
| TpDz | 4.552 | ^7^ |
| TD-COF | 4.06 | ^8^ |
| BTT-H3 COF | 1.588 | ^9^ |
| TaptBtt | 1.407 | ^10^ |
| TMB-COF-4 | 1.396 | ^11^ |
| sonoCOF-F2 | 1.24 | ^12^ |
| COF-N32 | 0.605 | ^13^ |

**Table S3**. Fitted decay lifetime values from the corresponding TRPL kinetic traces.*

| Photocatalyst | A_1_ | τ_1_ (ns) | A_2_ | τ_2_ (ns) | τ_ave_ (ns) | χ^2^ |
| --- | --- | --- | --- | --- | --- | --- |
| TPy-acr COF | 0.411 | 2.46 | 0.589 | 9.65 | 6.69 | 1.177 |
| TPy-imine COF | 0.597 | 0.678 | 0.403 | 2.70 | 1.49 | 1.167 |
| TPh-imine COF | 0.840 | 0.250 | 0.160 | 3.25 | 0.73 | 1.174 |

*Notes for the table: a bi-exponential decay model was used for the fitting process, as shown in the following equation, $I\left( t \right)=A_{1}\exp\left( -\frac{t}{\tau1} \right)+A_{2}\exp\left( -\frac{t}{\tau2} \right)$, where $I\left( t \right)$ is the intensity at time (t); τ_1_ and τ_2_ are fast and slow lifetime components, related to distinct recombination pathways; A_1_ and A_2_ are amplitude contributions, reflecting the relative weights of each decay component. Goodness-of-fit metrics (χ^2^) was used to validate the reliability of the fitting procedure. Amplitude-weighted average lifetime (τ_ave_) was calculated from the bi-exponential fitting of the TRPL decay curves using the following equation, $\tau_{\mathrm{av}e}=A_{1}\tau_{1}+A_{2}\tau_{2}$.

**References**

1 Y. Chen, R. Liu, Y. Guo, et al.*,* *"Hierarchical assembly of donor–acceptor covalent organic frameworks for photosynthesis of hydrogen peroxide from water and air*," *Nature Synthesis* 3, no. 8 (2024): 998-1010.

2 J.-Y. Yue, J.-X. Luo, Z.-X. Pan, et al.*,* "Phenanthridine-based covalent organic frameworks for boosting overall solar H_2_O_2_ production," *Angewandte Chemie International Edition* 64, no. 5 (2025): e202417115.

3 R. Sun, X. Yang, X. Hu, et al.*,* "Unprecedented photocatalytic hydrogen peroxide production via covalent triazine frameworks constructed from fused building blocks," *Angewandte Chemie International Edition* 64, no. 4 (2025): e202416350.

4 R. Liu, Y. Chen, H. Yu, et al.*,* "Linkage-engineered donor–acceptor covalent organic frameworks for optimal photosynthesis of hydrogen peroxide from water and air," *Nature Catalysis* 7, no. 2 (2024): 195-206.

5 J.-Y. Yue, J.-X. Luo, Z.-X. Pan, et al.*,* "Regulating the topology of covalent organic frameworks for boosting overall H_2_O_2_ photogeneration," *Angewandte Chemie International Edition* 63, no. 24 (2024): e202405763.

6 L. Zhang, C. Wang, Q. Jiang, P. Lyu, Y. Xu*,* "Structurally locked high-crystalline covalent triazine frameworks enable remarkable overall photosynthesis of hydrogen peroxide," *Journal of the American Chemical Society* 146, no. 43 (2024): 29943-29954.

7 Q. Liao, Q. Sun, H. Xu, et al.*,* "Regulating relative nitrogen locations of diazine functionalized covalent organic frameworks for overall H_2_O_2_ photosynthesis," *Angewandte Chemie International Edition* 62, no. 41 (2023): e202310556.

8 J.-Y. Yue, L.-P. Song, Y.-F. Fan, et al.*,* "Thiophene-containing covalent organic frameworks for overall photocatalytic H_2_O_2_ synthesis in water and seawater," *Angewandte Chemie International Edition* 62, no. 38 (2023): e202309624.

9 A. Chakraborty, A. Alam, U. Pal, et al.*,* "Enhancing photocatalytic hydrogen peroxide generation by tuning hydrazone linkage density in covalent organic frameworks," *Nature Communications* 16, no. 1 (2025): 503.

10 C. Qin, X. Wu, L. Tang, et al.*,* "Dual donor-acceptor covalent organic frameworks for hydrogen peroxide photosynthesis," *Nature Communications* 14, no. 1 (2023): 5238.

11 R.-M. Zhu, Y. Liu, W.-K. Han, et al.*,* "Three-dimensional covalent organic frameworks based on linear and trigonal linkers for high-performance H_2_O_2_ photosynthesis," *Angewandte Chemie International Edition* 64, no. 1 (2025): e202412890.

12 W. Zhao, P. Yan, B. Li, et al.*,* "Accelerated synthesis and discovery of covalent organic framework photocatalysts for hydrogen peroxide production," *Journal of the American Chemical Society* 144, no. 22 (2022): 9902-9909.

13 F. Liu, P. Zhou, Y. Hou, et al.*,* "Covalent organic frameworks for direct photosynthesis of hydrogen peroxide from water, air and sunlight," *Nature Communications* 14, no. 1 (2023): 4344.
